# Comparative effectiveness of ceftolozane/tazobactam relative to ceftazidime/avibactam for the treatment of adult, hospitalized patients with pneumonia due to multidrug-resistant *Pseudomonas aeruginosa* in U.S. Veterans Health Administration facilities

**DOI:** 10.1128/aac.00707-26

**Published:** 2026-09-09

**Authors:** Nicholas S. Britt, Karim Khader, Tao He, Emily M. Potter, Thomas P. Lodise

**Affiliations:** 1 Department of Pharmacy Practice, University of Kansas School of Pharmacy 12251, Lawrence, Kansas, USA; 2 Department of Internal Medicine, University of Kansas School of Medicine 12251, Kansas City, Kansas, USA; 3 Dwight D. Eisenhower Veterans Affairs Medical Center, Leavenworth, Kansas, USA; 4 Department of Internal Medicine, University of Utah School of Medicine, Salt Lake City, Utah, USA; 5 Veterans Affairs Salt Lake City Health Care System 20122 , Salt Lake City, Utah, USA; 6 Department of Pharmacy Practice, Albany College of Pharmacy and Health Sciences 1091, Albany, New York, USA; 7 Samuel S. Stratton Veterans Affairs Medical Center, Albany, New York, USA; University of Pennsylvania Perelman School of Medicine, Philadelphia, Pennsylvania, USA

**Keywords:** ceftazidime, avibactam, ceftolozane, tazobactam, beta-lactam, pneumonia, multidrug-resistance, *Pseudomonas aeruginosa*, antimicrobial stewardship, Veterans Health Administration

## Abstract

Ceftolozane/tazobactam (C/T) and ceftazidime/avibactam (C/A) are frequently used for the treatment of multidrug-resistant *Pseudomonas aeruginosa* (MDR-PSA) pneumonia. However, comparative “real-world” effectiveness data are limited. We performed a retrospective cohort study within the U.S. Veterans Health Administration (VHA; 2015–2019, 2022–2024), including adults with microbiologically confirmed MDR-PSA pneumonia receiving C/T or C/A within ≤5 days of index culture. Inverse probability of treatment weighting (IPTW) was applied to balance baseline covariates. The primary outcome was 30-day all-cause mortality, and secondary outcomes were 60-day mortality, 90-day mortality, and 30-, 60-, and 90-day MDR-PSA recurrence and all-cause hospital readmission. A prespecified sensitivity analysis examined the effect of early treatment initiation ≤72 h. Among 260 patients (C/T, *n* = 132; C/A, *n* = 128), C/T recipients demonstrated more extensive prior antimicrobial exposure and substantially longer time-to-therapy (median, 56 versus 19 h; *P* < 0.001). After IPTW, balance improved across most prespecified covariates, although residual imbalance persisted for age and geographic region. No statistically significant between-group differences were observed in 30-day mortality (adjusted risk ratio [aRR], 1.02; 95% confidence interval [CI], 0.56–1.83; *P* = 0.944) or in other clinical outcomes. Among patients with early treatment within 72 h (*n* = 175), no significant differences in 30-day mortality (aRR, 1.18; 95% CI, 0.47–2.93; *P* = 0.724) or other mortality, recurrence, or readmission outcomes were observed between C/T and C/A. In this national VHA cohort, C/T and C/A demonstrated comparable clinical, microbiologic, and healthcare utilization outcomes. These findings support consideration of either C/T or C/A for MDR-PSA pneumonia when used at appropriate doses.

## INTRODUCTION

Multidrug resistance (MDR) among *Pseudomonas aeruginosa* (PSA) presents a significant clinical challenge and can substantially complicate selection of optimal antibiotic therapy (1). Multidrug-resistant PSA remains one of the most clinically important pathogens encountered in hospital-acquired bacterial pneumonia (HABP) and ventilator-associated bacterial pneumonia (VABP) (2). Over the past decade, the increasing prevalence of MDR-PSA has further complicated empiric and definitive treatment selection in hospitalized patients, underscoring the need for additional comparative effectiveness data (3–5).

Novel β-lactam/β-lactamase inhibitor combinations, such as ceftolozane/tazobactam (C/T) and ceftazidime/avibactam (C/A), are increasingly used in clinical practice for the treatment of MDR-PSA pneumonia (4–7). These agents demonstrate enhanced activity against many MDR-PSA strains and are approved by the U.S. Food and Drug Administration for the treatment of HABP/VABP (8, 9). Despite increasing use, comparative real-world effectiveness data for C/T and C/A remain sparse, and prior studies have not consistently accounted for clinically important factors, such as appropriate dosing, coronavirus disease 2019 (COVID-19) infection, and timing of therapy initiation (6, 7, 10–20). As such, selection of optimal therapy for MDR-PSA pneumonia remains challenging, particularly in high-risk patients with extensive comorbidity burden, renal dysfunction, and critical illness.

Given these considerations, we conducted a national retrospective cohort study comparing the effectiveness of C/T versus C/A for the treatment of MDR-PSA pneumonia within the Veterans Health Administration (VHA), the largest integrated healthcare system in the United States. Our primary objective was to evaluate mortality, recurrence, and healthcare utilization outcomes between C/T and C/A for MDR-PSA pneumonia while accounting for baseline differences and treatment selection bias influencing clinical decision-making.

## RESULTS

After screening approximately 1.80 million inpatient VHA encounters, 260 patients from 66 hospitals met the study criteria for MDR-PSA pneumonia and were included in the primary analytic cohort (Table 1). Of these, 132 (50.8%) received C/T and 128 (49.2%) received C/A within 5 days of the index culture as definitive therapy. The median treatment duration was 7 days for both groups (C/T, IQR 5–13 days; C/A, IQR 4–12 days) and did not differ significantly between groups (*P* = 0.339).

**TABLE 1:** Study attrition

Eligibility criterion	Overall, *n*	C/A, *n*	C/T, *n*
Inpatient admission to a VHA facility from 1 January 2015 to 31 December 2019 or from 1 January 2022 to 31 December 2024	1,798,527		
Respiratory specimen positive for *P. aeruginosa*	19,088		
Respiratory specimen positive for MDR *P. aeruginosa*	5,379		
Radiographic evidence of pneumonia within 48 h of collection of the MDR-PSA respiratory specimen	4,746		
Receipt of C/A or C/T within 5 days of the index culture, with treatment continued for >2 calendar days^*a*^	372	187	218
First qualifying MDR-PSA pneumonia episode, with treatment classified according to the predominant study agent received^*b*^	369	167	202
Receipt of the U.S. FDA-recommended HABP/VABP dosage of C/A or C/T according to CrCl category by the second day of therapy, with CrCl ≥ 16 mL/min	281	137	144
Survival for >48 h after initiation of C/A or C/T	277	136	141
No recorded COVID-19 diagnosis within 90 days before the index MDR-PSA culture	260	128	132

^*a*^ Patients who received both C/A and C/T could be included in both treatment-specific columns at this step; therefore, the treatment-specific counts do not sum to the number of unique patients.

^*b*^ For patients who received both agents, treatment was classified according to the agent received for the greater number of treatment days.

Baseline characteristics were compared between treatment groups (Table 2). The majority of patients were elderly (median age, 70 years) and required intensive care unit (ICU) admission and mechanical ventilation. Notable baseline differences between treatment groups included longer time-to-therapy among those treated with C/T compared with C/A (median, 56 versus 19 h; *P* < 0.001), more frequent prior respiratory PSA isolation among C/T-treated patients versus C/A-treated patients (59.1% versus 43.8%; *P* = 0.013), and more frequent prior antibiotic exposure among C/T-treated patients versus C/A-treated patients (95.5% versus 88.3%; *P* = 0.034). Most patients had creatinine clearance (CrCl) > 50 mL/min and were treated with maximum optimized doses of C/T or C/A, although more patients treated with C/T had a prior diagnosis of chronic kidney disease compared with those treated with C/A (82.6% versus 71.1%; *P* = 0.028). There were no other statistically significant differences between groups with regard to baseline comorbidities, Charlson comorbidity index (CCI), or disease severity measures, such as systemic inflammatory response syndrome (SIRS) criteria and modified Acute Physiology and Chronic Health Evaluation (APACHE) II score (21).

**TABLE 2:** Comparison of baseline characteristics between hospitalized patients with multidrug-resistant *Pseudomonas aeruginosa* pneumonia treated with ceftazidime/avibactam versus ceftolozane/tazobactam^*a*^

Characteristic (*N*=260)	C/A (*n* = 128)	C/T (*n* = 132)	*P*
Age (years), median (Q1, Q3)	70 (63, 76)	70 (61, 76)	0.518
Age ≥ 65 years, n (%)	90 (70.3)	95 (72.0)	0.768
High complexity hospital, n (%)	117 (91.4)	126 (95.5)	0.187
Geographic region, n (%)			0.003
North Atlantic	12 (9.4)	22 (16.7)	
Southeast	30 (23.4)	52 (39.4)	
Midwest	63 (49.2)	42 (31.8)	
Continental	23 (18.0)	16 (12.1)	
Year of infection, *n* (%)			0.007
2015	0 (0.0)	5 (3.8)	
2016	8 (6.3)	7 (5.3)	
2017	12 (9.4)	4 (3.0)	
2018	23 (18.0)	17 (12.9)	
2019	19 (14.8)	18 (13.6)	
2022	29 (22.7)	26 (19.7)	
2023	10 (7.8)	29 (22.0)	
2024	27 (21.1)	26 (19.7)	
Male, *n* (%)	126 (98.4)	129 (97.7)	0.677
Obese, *n* (%)^*b*^	27 (21.1)	22 (16.7)	0.361
Mechanically ventilated, *n* (%)	91 (71.1)	100 (75.8)	0.394
ICU admission, *n* (%)	89 (69.5)	99 (75.0)	0.325
Hospital length of stay prior to culture (h) median (Q1, Q3)	55 (6, 327)	96 (1, 442)	0.762
Hospital-acquired, *n* (%)^*c*^	69 (53.9)	73 (55.3)	0.821
Hours from index culture to index treatment, median (Q1, Q3)	19 (0, 77)	56 (23, 80)	<0.001
Early treatment within 72 h, *n* (%)	89 (69.5)	86 (65.2)	0.452
Difficult-to-treat resistance (DTR) PSA, *n* (%)	11 (8.6)	17 (12.9)	0.265
Carbapenem-resistant (CR) PSA, *n* (%)	109 (85.2)	113 (85.6)	0.918
Polymicrobial pneumonia, any bacteria, *n* (%)	43 (33.6)	38 (28.8)	0.403
Polymicrobial pneumonia, excluding *Staphylococcus* spp. and *Streptococcus* spp., *n* (%)	41 (32.0)	35 (26.5)	0.328
SIRS criteria, *n* (%)			0.711
0	17 (13.3)	18 (13.6)	
1	44 (34.4)	50 (37.9)	
2	38 (29.7)	38 (28.8)	
3	26 (20.3)	20 (15.2)	
4	3 (2.3)	6 (4.5)	
Sepsis, *n* (%)^*d*^	97 (75.8)	99 (75.0)	0.884
Sepsis with evidence of tissue hypoperfusion, *n* (%)^*e*^	19 (14.8)	14 (10.6)	0.305
Vasopressor requirement, *n* (%)	40 (31.3)	35 (26.5)	0.400
Inodilator requirement, *n* (%)	0 (0.0)	1 (0.8)	0.324
Creatinine clearance, *n* (%)			0.214
>50 mL/min	86 (67.2)	88 (66.7)	
30–50 mL/min	15 (11.7)	24 (18.2)	
16–29 mL/min	27 (21.1)	20 (15.2)	
Acute kidney injury, *n* (%)^*f*^	54 (42.2)	57 (43.2)	0.871
Hospitalization previous 90 days, *n* (%)	62 (48.4)	74 (56.1)	0.219
Prior respiratory culture PSA positive during admission, *n* (%)^*g*^	56 (43.8)	78 (59.1)	0.013
Prior blood culture PSA positive during admission, *n* (%)^*g*^	10 (7.8)	13 (9.8)	0.563
Prior antibiotics received during admission, any, *n* (%)	113 (88.3)	126 (95.5)	0.034
Prior aminoglycoside received during admission, *n* (%)	38 (29.7)	42 (31.8)	0.710
Prior non-anti-PSA beta-lactam received during admission, *n* (%)	51 (39.8)	65 (49.2)	0.127
Prior anti-PSA beta-lactam received during admission, *n* (%)	104 (81.3)	120 (90.9)	0.024
Prior fluoroquinolone received during admission, *n* (%)	35 (27.3)	47 (35.6)	0.152
Prior vancomycin received during admission, *n* (%)	92 (71.9)	103 (78.0)	0.252
Prior daptomycin received during admission, *n* (%)	12 (9.4)	18 (13.6)	0.282
Prior macrolide received during admission, *n* (%)	21 (16.4)	23 (17.4)	0.827
Prior linezolid received during admission, *n* (%)	13 (10.2)	17 (12.9)	0.492
Prior tetracycline received during admission, *n* (%)	14 (10.9)	8 (6.1)	0.158
Prior sulfonamide received during admission, *n* (%)	13 (10.2)	20 (15.2)	0.226
Prior polymyxin received during admission, *n* (%)	10 (7.8)	3 (2.3)	0.040
Number of prior antibiotics received during admission, any, *n* (%)			0.198
0	15 (11.7)	6 (4.5)	
1	14 (10.9)	12 (9.1)	
2	13 (10.2)	20 (15.2)	
3	35 (27.3)	35 (26.5)	
4+	51 (39.8)	59 (44.7)	
Concomitant antibiotics received, any, *n* (%)^*h*^	113 (88.3)	120 (90.9)	0.487
Concomitant antibiotics received, anti-Psa, *n* (%)^*h*^	96 (75.0)	103 (78.0)	0.564
Concomitant aminoglycoside received, *n* (%)^*h*^	29 (22.7)	32 (24.2)	0.763
Concomitant non-anti-Psa beta-lactam received, *n* (%)^*h*^	6 (4.7)	19 (14.4)	0.008
Concomitant anti-Psa beta-lactam received, *n* (%)^*h*^	81 (63.3)	84 (63.6)	0.953
Concomitant fluoroquinolone received, *n* (%)^*h*^	18 (14.1)	20 (15.2)	0.804
Concomitant vancomycin received, *n* (%)^*h*^	69 (53.9)	63 (47.7)	0.319
Concomitant daptomycin received, *n* (%)^*h*^	8 (6.3)	9 (6.8)	0.853
Concomitant macrolide received, *n* (%)^*h*^	4 (3.1)	5 (3.8)	0.770
Concomitant linezolid received, *n* (%)^*h*^	6 (4.7)	9 (6.8)	0.461
Concomitant tetracycline received, *n* (%)^*h*^	7 (5.5)	3 (2.3)	0.180
Concomitant sulfonamide received, *n* (%)^*h*^	6 (4.7)	4 (3.0)	0.487
Concomitant polymyxin received, *n* (%)^*h*^	7 (5.5)	2 (1.5)	0.081
Number of concomitant antibiotics received, any, *n* (%)^*h*^			0.782
0	15 (11.7)	12 (9.1)	
1	33 (25.8)	35 (26.5)	
2	44 (34.4)	54 (40.9)	
3	25 (19.5)	21 (15.9)	
4+	11 (8.6)	10 (7.6)	
Confirmed susceptibility to C/T, *n* (%)	9 (7.0)	7 (5.3)	0.562
Confirmed susceptibility to C/A, *n* (%)	4 (3.1)	6 (4.5)	0.552
Peptic ulcer disease, *n* (%)	19 (14.8)	22 (16.7)	0.687
Rheumatologic disease, *n* (%)	4 (3.1)	5 (3.8)	0.770
Chronic kidney disease, *n* (%)	91 (71.1)	109 (82.6)	0.028
Peripheral vascular disease, *n* (%)	39 (30.5)	51 (38.6)	0.166
Hemiplegia/paraplegia, *n* (%)	50 (39.1)	55 (41.7)	0.669
Myocardial infarction, *n* (%)	32 (25.0)	32 (24.2)	0.887
Malignancy, any, *n* (%)	38 (29.7)	36 (27.3)	0.666
Metastatic solid tumor, *n* (%)	9 (7.0)	14 (10.6)	0.310
Liver disease, mild, *n* (%)	19 (14.8)	30 (22.7)	0.104
Liver disease, moderate-severe, *n* (%)	16 (12.5)	19 (14.4)	0.655
Dementia, *n* (%)	39 (30.5)	34 (25.8)	0.398
Diabetes, uncomplicated, *n* (%)	51 (39.8)	54 (40.9)	0.861
Diabetes, complicated, *n* (%)	45 (35.2)	50 (37.9)	0.649
Cerebrovascular disease, *n* (%)	52 (40.6)	50 (37.9)	0.650
Chronic obstructive pulmonary disease, *n* (%)	87 (68.0)	90 (68.2)	0.971
Congestive heart failure, *n* (%)	62 (48.4)	76 (57.6)	0.140
Human immunodeficiency virus infection, *n* (%)	2 (1.6)	1 (0.8)	0.543
Charlson comorbidity index, median (Q1, Q3)	9.0 (7.0, 12.0)	10.0 (7.5, 13.0)	0.088
Modified APACHE II score, median (Q1, Q3)	14.5 (11.0, 17.0)	14.0 (11.5, 18.0)	0.523

^*a*^ Continuous variables compared by Wilcoxon rank sum test and categorical variables compared by Pearson's chi-squared test or Fisher’s exact test.

^*b*^ Body mass index ≥ 30.

^*c*^ MDR-PSA culture positive >48 h after admission.

^*d*^ SIRS ≥ 2 or ICD-9/10-CM diagnosis code for sepsis.

^*e*^ Sepsis plus elevated lactate or systolic blood pressure < 90 mmHg.

^*f*^ Determined by ICD-9/10-CM diagnosis code.

^*g*^ Any culture positive for PSA collected before index MDR-PSA culture during the same hospitalization. Prior isolates were not required to meet MDR criteria or fulfill the study definition of pneumonia.

^*h*^ Receipt of ≥1 dose during the period from first C/T or C/A day of therapy for index MDR-PSA pneumonia until the end of treatment day of therapy with C/T or C/A.

Unadjusted mortality at 30 days was 19.2% (*n* = 50/260) overall and did not differ significantly between groups (16.4% versus 22.0%; risk ratio [RR], 1.34; 95% confidence interval [CI], 0.80–2.23; *P* = 0.260; Table 3). There were no significant differences in 60- or 90-day mortality endpoints between treatment groups. Recurrence of MDR-PSA pneumonia within 30 days was relatively common, occurring in 26.2% of patients (*n* = 68/260). Treatment with C/T did not significantly reduce the risk of 30-day MDR-PSA recurrence compared with C/A (27.3% versus 25.0%; RR, 1.10; 95% CI, 0.72–1.65; *P* = 0.678). No differences in 60- or 90-day MDR-PSA pneumonia recurrence, hospital readmission (30-, 60-, or 90-day), composite clinical success, hospital length of stay (LOS), or ICU-free days, were observed between groups in unadjusted analyses (Table 3).

**TABLE 3:** Comparison of clinical outcomes between hospitalized patients with multidrug-resistant *Pseudomonas aeruginosa* pneumonia treated with ceftazidime/avibactam versus ceftolozane/tazobactam

Outcome (*N* = 260)	C/A (*n* = 128)	C/T (*n* = 132)	Unadjusted RR (95% CI)	*P*	IPTW-Adjusted RR^*a*^ (95% CI)	*P*
All-cause mortality, *n* (%)						
30-day mortality	21 (16.4)	29 (22.0)	1.34 (0.80–2.23)	0.260	1.02 (0.56–1.83)	0.944
60-day mortality	38 (29.7)	44 (33.3)	1.12 (0.78–1.62)	0.529	0.99 (0.66–1.49)	0.977
90-day mortality	52 (40.6)	58 (43.9)	1.08 (0.81–1.44)	0.590	1.06 (0.77–1.47)	0.710
MDR-PSA pneumonia recurrence, *n* (%)						
30-day recurrence	32 (25.0)	36 (27.3)	1.10 (0.72–1.65)	0.678	1.19 (0.75–1.88)	0.460
60-day recurrence	41 (32.0)	44 (33.3)	1.04 (0.73–1.48)	0.824	1.09 (0.73–1.62)	0.672
90-day recurrence	43 (33.6)	45 (34.1)	1.01 (0.72–1.43)	0.933	1.05 (0.71–1.55)	0.796
All-cause hospital readmission, n (%)						
30-day readmission	22 (17.2)	21 (15.9)	0.93 (0.53–1.60)	0.782	1.10 (0.61–1.99)	0.755
60-day readmission	34 (26.6)	30 (22.7)	0.86 (0.55–1.31)	0.475	0.81 (0.50–1.30)	0.375
90-day readmission	37 (28.9)	33 (25.0)	0.86 (0.58–1.30)	0.480	0.83 (0.53–1.30)	0.407
Clinical success at 30 days, *n* (%)^*b*^	65 (50.8)	55 (41.7)	0.82 (0.63–1.07)	0.144	0.85 (0.62–1.14)	0.278
Hospital length of stay (days), median, IQR^*c*^	22 (11–47)	28.5 (14–71)		0.078		
ICU-free days, median (IQR)^*c*^ ^*d*^	19.4 (0–28)	11.1 (0–28)		0.125		

^*a*^ Propensity score models included the following variables: High complexity hospital, ICU admission, obesity, time to infection, vasopressor use, SIRS criteria, creatinine clearance category, prior PSA respiratory culture positive during admission, hospitalization in the previous 90 days, polymicrobial pneumonia, time to treatment, prior antibiotics during admission ≥4, Charlson comorbidity index, modified APACHE II score.

^*b*^ Composite of 30-day all-cause mortality, 30-day MDR-PSA pneumonia recurrence, and 30-day all-cause hospital readmission.

^*c*^ Compared by Wilcoxon rank sum test.

^*d*^ Within 30 days from start of treatment.

After propensity score derivation and inverse probability of treatment weighting (IPTW), covariate balance improved across most prespecified characteristics, although residual imbalance remained, most notably for age and geographic region (Fig. S1, Table S1) (22). In IPTW-adjusted Poisson regression analysis, there was no significant difference in 30-day mortality between groups (adjusted RR [aRR], 1.02; 95% CI, 0.56–1.83; *P* = 0.944). No significant differences between groups were observed for 60- or 90-day all-cause mortality, MDR-PSA pneumonia recurrence (30-, 60-, 90-day), hospital readmission (30-, 60-, or 90-day), composite clinical success, hospital LOS, or ICU-free days after IPTW adjustment (Table 3; Table S2). To further address potential confounding by geographic variation and temporal prescribing patterns, an alternative propensity score model additionally incorporating geographic region and year of infection was evaluated and improved balance across the modeled covariates (Fig. S2). IPTW-adjusted estimates from this model showed no statistically significant between-group differences in mortality, MDR-PSA pneumonia recurrence, hospital readmission, or 30-day clinical success, consistent with the primary analysis (Table S3).

Among patients who experienced MDR-PSA pneumonia recurrence, the median time to recurrence was 10 days (IQR, 4–25 days) for C/T-treated patients and 16 days (IQR, 8–31 days) for C/A-treated patients (*P* = 0.094). Kaplan–Meier analysis showed no statistically significant between-group difference in time to recurrence (log-rank *P* = 0.661; Fig. S3A). Similarly, cause-specific Cox regression, with patients censored at death, showed no statistically significant association between treatment and recurrence in either the unadjusted analysis (HR, 1.10; 95% CI, 0.72–1.67; *P* = 0.663) or the IPTW-adjusted analysis (aHR, 1.12; 95% CI, 0.71–1.77; *P* = 0.626). However, the proportional hazards assumption was not met for the IPTW-adjusted model (Schoenfeld test, *P* = 0.005). Therefore, a time-varying coefficient analysis was performed and demonstrated a decreasing relative hazard associated with C/T treatment over time (treatment-by-time interaction *P* = 0.023). The estimated recurrence hazard was significantly higher with C/T at day 4 (aHR, 1.89; 95% CI, 1.03-3.47; *P* = 0.040), but the association attenuated thereafter and point estimates favored C/T after day 30 (Table S4).

Among patients who were readmitted within 90 days, the median time to all-cause hospital readmission was 23 days (IQR, 13–42 days) in the C/T group and 24 days (IQR, 11–37 days) in the C/A group (*P* = 0.920). Kaplan–Meier analysis, with death treated as a censoring event, showed no statistically significant between-group difference in time to readmission (log-rank *P* = 0.575; Fig. S3B). Similarly, cause-specific Cox regression showed no statistically significant association between treatment and readmission in either the unadjusted analysis (HR, 0.88; 95% CI, 0.55–1.40; *P* = 0.576) or the IPTW-adjusted analysis (aHR, 0.84; 95% CI, 0.50–1.41; *P* = 0.515). In competing-risk analyses using Aalen–Johansen cumulative incidence functions, no statistically significant between-group differences were observed in MDR-PSA pneumonia recurrence (Gray’s *P* = 0.079; Fig. S4A) or all-cause hospital readmission (Gray’s *P* = 0.473; Fig. S4B).

### Subgroup and sensitivity analyses

In secondary analyses, there were no significant differences between unadjusted 30-day mortality stratified by elderly age, hospital-acquired infection, ICU admission, carbapenem-resistant (CR)-PSA, difficult-to-treat resistant (DTR)-PSA, polymicrobial pneumonia, concomitant aminoglycoside therapy, prior PSA-positive respiratory culture during admission, and period of infection (Table 4). In a sensitivity analysis limiting the cohort to those treated with C/T or C/A within 72 h of the index MDR-PSA respiratory culture (*n* = 175), baseline characteristics were compared between groups (Table S5). Notable differences between groups at baseline included longer median time-to-therapy (33 h versus 8 h; *P* < 0.001), more frequent prior hospitalization within 90 days (60.5% versus 44.9%; *P* = 0.040), prior respiratory culture positive with PSA during admission (67.4% versus 48.3%; *P* = 0.010), prior antibiotics received during admission (95.3% versus 85.4%; *P* = 0.026), prior anti-PSA β-lactam antibiotics received during admission (89.5% versus 75.3%; *P* = 0.014), and higher median CCI (11.0 versus 9.0; *P* = 0.044) in C/T-treated patients compared with C/A (23). There was numerically higher 30-day mortality (20.9% versus 10.1%; RR, 2.07; 95% CI, 0.97–4.38; *P* = 0.057) among C/T recipients in the early treatment subgroup; however, this did not reach the threshold for statistical significance (Table S6). No statistically significant differences between groups were observed for any of the other clinical outcomes measured. After IPTW, baseline covariates were relatively well-balanced between groups, with some diminished overlap in time-to-therapy and CrCl category (Fig. S5). In the early treatment subgroup, IPTW-adjusted analyses demonstrated no significant differences between C/T and C/A for 30-day mortality (aRR, 1.18; 95% CI, 0.47–2.93; *P* = 0.724), 60-day mortality (aRR, 1.15; 95% CI, 0.61–2.16; *P* = 0.666), or 90-day mortality (aRR, 0.92; 95% CI, 0.57–1.48; *P* = 0.724). Other clinical outcomes, including MDR-PSA recurrence, hospital readmission, composite clinical success, hospital LOS, and ICU-free days were similar between groups, with no statistically significant differences observed at any time point assessed (Table S6).

**TABLE 4:** Secondary stratified analyses of 30-day all-cause mortality among hospitalized patients with multidrug-resistant *Pseudomonas aeruginosa* pneumonia treated with ceftazidime/avibactam versus ceftolozane/tazobactam^*a*^

Stratum (*N* = 260)	Unadjusted RR (95% CI)	*P*
Elderly		
Age ≥ 65 years (*n* = 185)	1.10 (0.63–1.90)	0.739
Age < 65 years (*n* = 75)	3.60 (0.77–16.8)	0.102
Hospital-acquired^*b*^		
Yes (*n* = 142)	1.16 (0.60–2.25)	0.652
No (*n* = 118)	1.63 (0.72–3.67)	0.241
ICU admission		
Yes (*n* = 188)	1.27 (0.76–2.14)	0.351
No (*n* = 72)	1.18 (0.17–8.32)	0.865
Carbapenem-resistant		
Yes (*n* = 222)	1.54 (0.85-2.80)	0.151
No (*n* = 38)	0.83 (0.29-2.38)	0.730
Difficult-to-treat resistant		
Yes (*n* = 28)	0.33 (0.02–3.68)	0.359
No (*n* = 232)	1.50 (0.89–2.53)	0.131
Polymicrobial pneumonia		
Yes (*n* = 81)	1.32 (0.48–3.67)	0.590
No (*n* = 179)	1.33 (0.73–2.40)	0.348
Concomitant aminoglycoside therapy^*c*^		
Yes (*n* = 61)	1.06 (0.39–2.86)	0.911
No (*n* = 199)	1.45 (0.80–2.65)	0.222
Prior positive PSA respiratory culture positive during admission^*d*^		
Yes (*n* = 134)	1.71 (0.80–3.65)	0.168
No (*n* = 126)	1.03 (0.48–2.18)	0.947
Period of infection		
2015–2019 (*n* = 113)	1.76 (0.81–3.81)	0.154
2022–2024 (*n* = 147)	1.09 (0.55–2.15)	0.811

^*a*^ Risk ratio >1 indicates increased 30-day all-cause mortality associated with C/T treatment.

^*b*^ MDR-PSA culture positive >48 h after admission.

^*c*^ Receipt of ≥1 dose of aminoglycoside during the period from first C/T or C/A day of therapy for index MDR-PSA pneumonia until the end of treatment day of therapy with C/T or C/A.

^*d*^ Any respiratory culture positive for PSA collected before index MDR-PSA culture during the same hospitalization. Prior isolates were not required to meet MDR criteria or fulfill the study definition of pneumonia.

Sequential exposure to both C/T and C/A during the index hospitalization occurred in 27 patients. Among the 132 patients classified as C/T-treated, 20 (15.2%) had previously received C/A, whereas 7 of the 128 patients classified as C/A-treated (5.5%) had previously received C/T (*P* = 0.011). In a post-hoc sensitivity analysis excluding these patients, 233 patients remained (C/A, *n* = 121; C/T, *n* = 112; Table S7). No statistically significant between-group differences were observed in the unadjusted analyses of mortality, MDR-PSA pneumonia recurrence, hospital readmission, 30-day clinical success, hospital length of stay, or ICU-free days (Table S8). A separate propensity score model was derived for the restricted cohort, and IPTW improved balance across the modeled covariates (Fig. S6). IPTW-adjusted analyses likewise showed no statistically significant differences in the assessed outcomes between C/T- and C/A-treated patients who did not receive sequential therapy (Table S8).

## DISCUSSION

In this national retrospective cohort study of hospitalized Veterans meeting the study definition for MDR-PSA pneumonia, we compared outcomes among patients treated with C/T or C/A. In the primary analyses, no statistically significant between-group differences were observed in all-cause mortality, MDR-PSA pneumonia recurrence, hospital readmission, composite clinical success, hospital length of stay, or ICU-free days, either before or after IPTW adjustment. These findings suggest similar observed effectiveness of C/T and C/A for MDR-PSA pneumonia.

Findings from the present study differ from some recently published analyses suggesting improved outcomes with C/T compared with C/A for MDR-PSA infections (15, 16). In the multicenter CACTUS study, C/T was associated with higher composite clinical success, driven largely by lower recurrence, without a significant difference in mortality (15). Similarly, an analysis of non-COVID-19 hospitalizations in the PINC AI Healthcare Database found that patients treated with C/T were approximately four times more likely to be discharged home alive without an unfavorable infection-related outcome, a composite that included pneumonia recurrence, in-hospital mortality, discharge to another healthcare facility, and pneumonia-related 30-day readmission (16). Conversely, several smaller retrospective studies and a recent network meta-analysis reported no significant differences between C/T and C/A, consistent with the primary findings of the present study (12, 17, 19, 20). Collectively, these disparate findings suggest that observed comparative effectiveness may vary according to the study population, outcome definitions, follow-up, and analytic approach.

Recurrence warrants particular consideration because absolute rates in the present study were higher than those reported in several previous analyses, although no statistically significant differences were observed between treatment groups in either the primary or competing-risk analyses. By contrast, CACTUS reported lower recurrence with C/T at both 30 and 90 days among patients with pneumonia, which contributed substantially to the observed difference in composite clinical success (15). Other analyses have similarly reported fewer recurrent MDR-PSA pneumonia events with C/T during the index hospitalization (16). Among patients in the present study who initiated therapy within 72 h, recurrence was numerically lower with C/T, although the differences were modest and imprecise. An exploratory time-varying analysis suggested that the association between treatment and recurrence may have changed over follow-up, with a higher estimated hazard with C/T early and attenuation thereafter. However, this post hoc finding should be interpreted cautiously. Overall, the recurrence difference observed in the early-treatment subgroup was smaller than that reported among patients with pneumonia in CACTUS. Differences in patient characteristics, recurrence definitions, follow-up, and microbiologic surveillance may contribute to variation across studies. The older and more medically complex VHA population, together with longitudinal follow-up within an integrated healthcare system (38), may also have increased both the underlying risk and ascertainment of recurrence, although these explanations remain speculative.

Variation in the timing of appropriate antimicrobial therapy may also contribute to heterogeneity across studies. Delays in receipt of active therapy have been consistently associated with worse outcomes in severe Gram-negative infections, including MDR-PSA pneumonia (24–26). In the present cohort, the median interval from index culture collection to initiation of study therapy was longer among patients treated with C/T than among those treated with C/A. This difference may reflect treatment-selection practices rather than a differential treatment effect. C/A may have been selected more frequently as empiric therapy because of its broader activity against resistant Gram-negative pathogens, whereas C/T may have been reserved for targeted therapy following microbiologic confirmation of MDR-PSA. Although IPTW was used to adjust for measured differences, some residual confounding related to treatment timing and treatment sequencing may have persisted. In the prespecified sensitivity analysis restricted to patients treated within 72 h, IPTW-adjusted analyses again identified no statistically significant differences between treatment groups. However, some residual imbalance remained in time to therapy and renal function, and the smaller sample size resulted in wide confidence intervals and limited precision. Thus, restricting the analysis to patients treated within 72 h did not materially alter the overall findings, although clinically important differences between treatments cannot be excluded. These findings underscore the importance of treatment timing and clinical context when interpreting comparative effectiveness estimates from observational data.

In addition to treatment timing, differences in study period and healthcare delivery may contribute to variability across studies. Several prior studies, including CACTUS, included a substantial proportion of patients hospitalized during the COVID-19 pandemic, a period associated with increased all-cause mortality among hospitalized patients (15, 27–29). This period was also characterized by system-level healthcare disruptions, including ICU strain, altered admission thresholds, staffing challenges, delayed diagnostics, and changes in antimicrobial utilization (27, 28). It also coincided with documented national shortages and intermittent availability of C/T, during which C/A may have been used more frequently for the treatment of MDR-PSA pneumonia at some centers (30–32). In the present study, admissions during 2020–2021 and patients with COVID-19 infection within 90 days before the index culture were excluded to reduce the influence of these factors. Although treatment selection varied over time, measured temporal and geographic differences did not appear to materially influence the comparative findings, which remained consistent with the primary analysis. Similarly, no statistically significant difference in 30-day mortality was observed between C/T and C/A within either the 2015–2019 or 2022–2024 period. Collectively, these findings suggest that measured temporal and geographic factors did not materially alter the observed treatment comparisons, although residual confounding related to evolving prescribing practices and healthcare delivery cannot be excluded (31, 32).

This study has several important strengths. It represents one of the largest comparative effectiveness studies of C/T and C/A for MDR-PSA reported to date. By leveraging the national scope of the VHA, this study enabled the inclusion of a geographically diverse and clinically complex patient population. Strict inclusion and exclusion criteria limited the cohort to those treated with appropriately dosed C/T or C/A and reduced the potential influence of COVID-19-related healthcare disruptions and the national C/T shortage. Through application of IPTW, we adjusted for measured sources of confounding, and multiple subgroup and sensitivity analyses were performed to assess the consistency of the findings across clinically relevant populations and analytic assumptions (22). Because standardized clinical signs and symptoms were not consistently available, pneumonia ascertainment was based on MDR-PSA isolation from a respiratory specimen, radiographic findings identified using a validated natural language processing (NLP) algorithm (33, 34), and clinician-directed antimicrobial treatment. This case definition was more specific than relying on respiratory cultures or administrative diagnosis codes alone, although it remains possible some patients with respiratory colonization may still have been misclassified as having pneumonia.

Several limitations should also be acknowledged. As a retrospective observational study, residual confounding cannot be excluded despite adjustment for measured covariates, particularly with respect to treatment timing, unmeasured markers of illness severity, and variability in antimicrobial stewardship and prescribing practices. Although admissions during the peak COVID-19 period were excluded, healthcare disruptions and temporal changes in prescribing may have persisted beyond this period and influenced treatment selection and outcomes (28, 35, 36). The VHA population is predominantly older and male, which may limit generalizability to other patient populations and healthcare settings (37). The NLP algorithm classified radiographic findings consistent with pneumonia and was not organism-specific (33, 34); attribution to MDR-PSA was established separately through respiratory culture criteria. Although the algorithm required contemporaneous radiographic reports describing new, progressive, or persistent pulmonary infiltrates within 48 h of the index MDR-PSA culture, persistent imaging abnormalities following an earlier pneumonia episode cannot be completely excluded. Differences in recurrence definitions, ascertainment methods, and follow-up periods also complicate direct comparison of absolute recurrence rates across studies of C/T and C/A. Finally, genomic resistance mechanisms and longitudinal susceptibility data were not uniformly available, precluding evaluation of treatment effects across specific molecular resistance profiles, emergence of resistance during therapy, and potential effect modification by active concomitant anti-PSA treatment (20).

In summary, C/T and C/A were associated with similar observed clinical, microbiologic, and healthcare utilization outcomes among patients with MDR-PSA pneumonia. Although these results should be taken within the context and limitations of the study design, the present study supports consideration of either agent when administered at optimized doses. Additional studies are needed to identify patient-specific and microbiologic factors that may influence comparative effectiveness and guide individualized treatment of these difficult-to-treat infections.

## MATERIALS AND METHODS

### Study design and population

We performed a retrospective, observational, multicenter cohort study of adult patients treated within the VHA. Data were extracted from the Veterans Affairs Corporate Data Warehouse (CDW), a comprehensive national repository that includes detailed demographics, inpatient and outpatient encounters, laboratory results, microbiologic cultures and susceptibility tests, radiology reports, vital status, and pharmacy records, including barcode medication administration timestamps (38). All analyses were performed within the Veterans Affairs Informatics and Computing Infrastructure (VINCI).

Patients were eligible for inclusion if they were hospitalized in a VHA facility between 1 January 2015 and 31 December 2019 or 1 January 2022 and 31 December 2024. Admissions during the 2020–2021 period were specifically excluded due to concerns for bias associated with the COVID-19 pandemic and a national drug shortage of C/T, which limited its use during a period of marked increases in all-cause mortality in hospitalized patients (27–32). Additional inclusion criteria were (i) age ≥18 years; (ii) isolation of PSA from a respiratory specimen meeting the NHSN definition for MDR based on facility-specific susceptibility testing (39, 40); (iii) clinical evidence of pneumonia, defined as new, progressive, or persistent pulmonary infiltrate, consolidation, or cavitation identified using a previously validated NLP algorithm of radiology reports collected within 48 h of MDR-PSA isolation (33, 34); (iv) receipt of either C/T or C/A within 5 days of MDR-PSA isolation and continuation of therapy for >2 calendar days; and (v) appropriate dosing of C/T or C/A for HABP/VABP based on CrCl category by day 2 of therapy (8, 9). The NLP algorithm identified radiographic findings consistent with pneumonia and was not organism-specific; microbiologic attribution to MDR-PSA was established separately through the respiratory culture criteria (33, 34). For C/T-treated patients, appropriate dosing was defined as: 3 g intravenous (IV) every 8 h for CrCl >50 mL/min, 1.5 g IV every 8 h for CrCl 30–50 mL/min, or 750 mg IV every 8 h for CrCl 16–29 mL/min (9). For C/A-treated patients, appropriate dosing regimens were defined as 2.5 g IV every 8 h for CrCl >50 mL/min, 1.25 g IV every 8 h for CrCl 30–50 mL/min, or 940 mg IV every 12 h for CrCl 16–29 mL/min (8). Patients were excluded if they (i) died within 48 h of treatment initiation; (ii) had severe renal dysfunction, defined as CrCl <16 mL/min or receipt of renal replacement therapy, including hemodialysis, at treatment initiation; or (iii) had recorded COVID-19 infection within 90 days before the index MDR-PSA culture. For patients receiving both C/T and C/A according to these criteria, exposure was classified according to the agent received for the greater number of treatment days.

### Baseline data collection

Baseline covariates were collected to reflect patient demographics, hospital characteristics, comorbid conditions, clinical severity, microbiologic factors, and antimicrobial exposures. Demographic variables included age, sex, and obesity. Comorbidities were assessed using International Classification of Disease 9th/10th Revision Clinical Modification (ICD-9/10-CM) codes, and comorbidity burden was measured according to the CCI (23). Markers of clinical severity at the time of index MDR-PSA culture included ICU admission status, mechanical ventilation, SIRS criteria, vasopressor or inotrope use, modified APACHE II score, and renal function category based on CrCl (21). Healthcare exposure variables included hospital LOS prior to index MDR-PSA culture collection and hospitalization within the preceding 90 days. Microbiologic variables included prior isolation of PSA during the index admission and the presence of polymicrobial pneumonia. Antimicrobial exposure variables captured the number and class of antibiotics, including prior anti-PSA therapy, received during admission before C/T or C/A initiation. Hospital-level characteristics included facility complexity designation and geographic region.

### Outcomes

Clinical outcomes evaluated were all-cause mortality at 30, 60, and 90 days. Microbiologic outcomes included MDR-PSA pneumonia recurrence at 30, 60, and 90 days. Recurrent MDR-PSA pneumonia was defined by all of the following criteria: (i) a subsequent respiratory culture positive for MDR-PSA within 90 days after completion of the initial C/T or C/A treatment course and following a treatment-free interval of >3 days, (ii) radiographic evidence of pneumonia identified by the NLP algorithm within 48 h of the subsequent MDR-PSA culture, and (iii) re-initiation of anti-PSA therapy. Healthcare utilization outcomes included hospital LOS from treatment initiation to discharge, ICU-free days within 30 days after treatment initiation, and all-cause hospital readmission within 30, 60, and 90 days (41, 42). Clinical success was evaluated as a composite endpoint requiring survival without recurrent MDR-PSA pneumonia or all-cause hospital readmission within 30 days.

### Statistical analysis

Baseline demographic, clinical, microbiologic, and hospital-level characteristics were compared using standard descriptive statistics. Continuous variables were compared between treatment groups using the Student’s *t*-test or Wilcoxon rank-sum test, as appropriate based on their distributions. Categorical variables were presented as frequencies and percentages and compared using Pearson’s chi-squared test or Fisher’s exact test. To address baseline covariate imbalances between C/T- and C/A-treated patients, IPTW was applied to estimate the average treatment effect (22). The probability of receiving C/T versus C/A was estimated using a logistic regression propensity score model, with covariates selected *a priori* based on clinical relevance, established predictors of poor outcomes in MDR-PSA pneumonia, and potential influence on clinical decision-making. Covariate balance was assessed using standardized mean differences (SMDs), with an absolute SMD < 0.1 considered indicative of adequate balance. Poisson regression models with robust variance estimators and IPTW were then used to estimate adjusted risk ratios for dichotomous outcomes. Time to MDR-PSA pneumonia recurrence and time to readmission were also evaluated using the Kaplan-Meier method and log-rank test and cause-specific Cox regression, with patients censored at death in both analyses. The proportional hazards assumption was evaluated using Schoenfeld residuals. When the proportional hazards assumption was not met, a time-varying coefficient sensitivity analysis incorporating a treatment-by-time interaction was performed, with time-specific aHRs estimated at clinically relevant follow-up intervals. Observed cumulative incidence in the presence of the competing risk of death was estimated using the Aalen-Johansen method, and treatment groups were compared using Gray’s test.

The association between treatment and 30-day mortality was also evaluated across subgroups defined by age ≥65 years, hospital-acquired infection, ICU admission, CR-PSA, DTR-PSA, polymicrobial pneumonia, concomitant aminoglycoside therapy, prior PSA-positive respiratory culture during the index hospitalization, and period of infection (2015–2019 versus 2022–2024). Given the established association between timely active therapy and outcomes in MDR-PSA pneumonia, a prespecified sensitivity analysis was restricted to patients who initiated C/T or C/A within 72 h of the index MDR-PSA respiratory culture (24–26). Additionally, given the observed sequential exposure to C/T and C/A, a post-hoc sensitivity analysis excluded patients who received both study agents sequentially during the index hospitalization. To evaluate the potential influence of geographic variation and temporal prescribing patterns, an alternative propensity score model incorporating geographic region and year of infection was also evaluated. Propensity score models were estimated separately within each restricted cohort, and covariate balance was reassessed using SMDs. Dichotomous outcomes were compared using IPTW-adjusted Poisson regression with robust variance estimators. All analyses were performed using R version 4.4.2 (R Foundation for Statistical Computing, Vienna, Austria). All statistical tests were two-sided, and *P* < 0.05 was considered statistically significant.

## Data Availability

The patient-level data used in this study were obtained from the Veterans Affairs Corporate Data Warehouse and analyzed within the Veterans Affairs Informatics and Computing Infrastructure. Because these data contain protected health information and are subject to U.S. Department of Veterans Affairs privacy and data security requirements, they cannot be deposited in a public repository. Access may be requested by qualified investigators through VINCI at VINCI@va.gov, and is subject to applicable VHA approvals and data-use permissions. Analytic code may be made available upon reasonable request and subject to VHA approval.

## SUPPLEMENTAL MATERIAL

Supplemental material aac.00707-26-s0001.docx Fig. S1 to S6; Tables S1 to S8.

ASM does not own the copyrights to Supplemental Material that may be linked to, or accessed through, an article. The authors have granted ASM a non-exclusive, world-wide license to publish the Supplemental Material files. Please contact the corresponding author directly for reuse.

## References

[1] Weiner LM, Webb AK, Limbago B, Dudeck MA, Patel J, Kallen AJ, Edwards JR, Sievert DM. 2016. Antimicrobial-resistant pathogens associated with healthcare-associated infections: summary of data reported to the national healthcare safety network at the centers for disease control and prevention, 2011–2014. Infect Control Hosp Epidemiol 37:1288–1301. doi:10.1017/ice.2016.174 PMC6857725 27573805

[2] Magill SS, Edwards JR, Bamberg W, Beldavs ZG, Dumyati G, Kainer MA, Lynfield R, Maloney M, McAllister-Hollod L, Nadle J, Ray SM, Thompson DL, Wilson LE, Fridkin SK, Emerging Infections Program Healthcare-Associated Infections and Antimicrobial Use Prevalence Survey Team. 2014. Multistate point-prevalence survey of health care-associated infections. N Engl J Med 370:1198–1208. doi:10.1056/NEJMoa1306801 PMC4648343 24670166

[3] Kadri SS. 2020. Key takeaways from the U.S. CDC’s 2019 antibiotic resistance threats report for frontline providers. Crit Care Med 48:939–945. doi:10.1097/CCM.0000000000004371 PMC7176261 32282351

[4] Paul M, Carrara E, Retamar P, Tängdén T, Bitterman R, Bonomo RA, de Waele J, Daikos GL, Akova M, Harbarth S, Pulcini C, Garnacho-Montero J, Seme K, Tumbarello M, Lindemann PC, Gandra S, Yu Y, Bassetti M, Mouton JW, Tacconelli E, Rodríguez-Baño J. 2022. European society of clinical microbiology and infectious diseases (ESCMID) guidelines for the treatment of infections caused by multidrug-resistant Gram-negative bacilli (endorsed by European society of intensive care medicine). Clin Microbiol Infect 28:521–547. doi:10.1016/j.cmi.2021.11.025 34923128

[5] Tamma PD, Aitken SL, Bonomo RA, Mathers AJ, van Duin D, Clancy CJ. 2022. Infectious diseases society of America 2022 guidance on the treatment of extended-spectrum β-lactamase producing *Enterobacterales* (ESBL-E), carbapenem-resistant enterobacterales (CRE), and *Pseudomonas aeruginosa* with difficult-to-treat resistance (DTR-P. aeruginosa). Clin Infect Dis 75:187–212. doi:10.1093/cid/ciac268doi:PMC9890506 35439291

[6] Puzniak L, Dillon R, Palmer T, Collings H, Enstone A. 2021. Real-world use of ceftolozane/tazobactam: a systematic literature review. Antimicrob Resist Infect Control 10:68. doi:10.1186/s13756-021-00933-8 PMC8027296 33832545

[7] Zhong H, Zhao XY, Zhang ZL, Gu ZC, Zhang C, Gao Y, Cui M. 2018. Evaluation of the efficacy and safety of ceftazidime/avibactam in the treatment of Gram-negative bacterial infections: a systematic review and meta-analysis. Int J Antimicrob Agents 52:443–450. doi:10.1016/j.ijantimicag.2018.07.004 30012440

[8] Torres A, Zhong N, Pachl J, Timsit JF, Kollef M, Chen Z, Song J, Taylor D, Laud PJ, Stone GG, Chow JW. 2018. Ceftazidime-avibactam versus meropenem in nosocomial pneumonia, including ventilator-associated pneumonia (REPROVE): a randomised, double-blind, phase 3 non-inferiority trial. Lancet Infect Dis 18:285–295. doi:10.1016/S1473-3099(17)30747-8 29254862

[9] Kollef MH, Nováček M, Kivistik Ü, Réa-Neto Á, Shime N, Martin-Loeches I, Timsit J-F, Wunderink RG, Bruno CJ, Huntington JA, Lin G, Yu B, Butterton JR, Rhee EG. 2019. Ceftolozane–tazobactam versus meropenem for treatment of nosocomial pneumonia (ASPECT-NP): a randomised, controlled, double-blind, phase 3, non-inferiority trial. Lancet Infect Dis 19:1299–1311. doi:10.1016/S1473-3099(19)30403-7 31563344

[10] Pogue JM, Kaye KS, Veve MP, Patel TS, Gerlach AT, Davis SL, Puzniak LA, File TM, Olson S, Dhar S, Bonomo RA, Perez F. 2020. Ceftolozane/tazobactam vs polymyxin or aminoglycoside-based regimens for the treatment of drug-resistant *Pseudomonas aeruginosa*. Clin Infect Dis 71:304–310. doi:10.1093/cid/ciz816 31545346

[11] Chen J, Liang Q, Chen X, Wu J, Wu Y, Teng G, Huang M. 2022. Ceftazidime/avibactam versus polymyxin B in the challenge of carbapenem-resistant *Pseudomonas aeruginosa* infection. Infect Drug Resist 15:655–667. doi:10.2147/IDR.S350976 PMC8887910 35241917

[12] Vena A, Giacobbe DR, Mussini C, Cattelan A, Bassetti M, Ceftabuse Study G. 2020. Clinical efficacy of ceftolozane-tazobactam versus other active agents for the treatment of bacteremia and nosocomial pneumonia due to drug-resistant *Pseudomonas aeruginosa*. Clin Infect Dis 71:1799–1801. doi:10.1093/cid/ciaa003 31904819

[13] Caffrey AR, Appaneal HJ, Liao JX, Piehl EC, Lopes V, Dillon RJ, Puzniak LA, LaPlante KL. 2022. The comparative effectiveness of ceftolozane/tazobactam versus aminoglycoside- or polymyxin-based regimens in multi-drug-resistant *Pseudomonas aeruginosa* infections. Antibiotics 11:626. doi:10.3390/antibiotics11050626 PMC9137796 35625270

[14] Qu J, Xu J, Liu Y, Hu C, Zhong C, Lv X. 2023. Real-world effectiveness of ceftazidime/avibactam versus polymyxin B in treating patients with carbapenem-resistant Gram-negative bacterial infections. Int J Antimicrob Agents 62:106872. doi:10.1016/j.ijantimicag.2023.106872 37247645

[15] Shields RK, Abbo LM, Ackley R, Aitken SL, Albrecht B, Babiker A, Burgoon R, Cifuentes R, Claeys KC, Curry BN, et al.. 2025. Effectiveness of ceftazidime-avibactam versus ceftolozane-tazobactam for multidrug-resistant *Pseudomonas aeruginosa* infections in the USA (CACTUS): a multicentre, retrospective, observational study. Lancet Infect Dis 25:574–584. doi:10.1016/S1473-3099(24)00648-0 39701120

[16] Lodise TP, Obi EN, Watanabe AH, Yucel E, Min J, Nathanson BH. 2024. Comparative evaluation of early treatment with ceftolozane/tazobactam versus ceftazidime/avibactam for non-COVID-19 patients with pneumonia due to multidrug-resistant *Pseudomonas aeruginosa*. J Antimicrob Chemother 79:2954–2964. doi:10.1093/jac/dkae313 PMC11531822 39258877

[17] Almangour TA, Ghonem L, Alassiri D, Aljurbua A, Al Musawa M, Alharbi A, Almohaizeie A, Almuhisen S, Alghaith J, Damfu N, Aljefri D, Alfahad W, Khormi Y, Alanazi MQ, Alsowaida YS. 2023. Ceftolozane-tazobactam versus ceftazidime-avibactam for the treatment of infections caused by multidrug-resistant *Pseudomonas aeruginosa*: a multicenter cohort study. Antimicrob Agents Chemother 67:e0040523. doi:10.1128/aac.00405-23 PMC10433809 37404159

[18] Collings H, Stone M, Chrysostomou M, Hirst A, Waldenberg TE, Yucel E, Iodise T. 2026. A systematic literature review and (network) meta-analysis of the effectiveness of ceftolozane/tazobactam versus aminoglycosides/polymyxins and ceftazidime/avibactam for treating adult patients with multidrug-resistant *Pseudomonas aeruginosa* infections. Antimicrob Agents Chemother 70:e0073525. doi:10.1128/aac.00735-25 PMC12888884 41532768

[19] El Nekidy WS, Al Ali M, Abidi E, El Lababidi R, Alrahmany D, Ghazi IM, Mooty M, Hijazi F, Ghosn M, Mallat J. 2024. Clinical outcomes of ceftazidime–avibactam versus ceftolozane–tazobactam in managing pseudomonal infections in patients undergoing renal replacement therapy. Antibiotics 13:699. doi:10.3390/antibiotics13080699 PMC11350821 39199999

[20] Hareza DA, Cosgrove SE, Bonomo RA, Dzintars K, Karaba SM, Hawes AM, Tekle T, Simner PJ, Tamma PD. 2024. Clinical outcomes and emergence of resistance of *Pseudomonas aeruginosa* infections treated with ceftolozane-tazobactam versus ceftazidime-avibactam. Antimicrob Agents Chemother 68:e0090724. doi:10.1128/aac.00907-24 PMC11459953 39230311

[21] Kollef KE, Reichley RM, Micek ST, Kollef MH. 2008. The modified APACHE II score outperforms Curb65 pneumonia severity score as a predictor of 30-day mortality in patients with methicillin-resistant *Staphylococcus aureus* pneumonia. Chest 133:363–369. doi:10.1378/chest.07-1825 17951615

[22] Chesnaye NC, Stel VS, Tripepi G, Dekker FW, Fu EL, Zoccali C, Jager KJ. 2022. An introduction to inverse probability of treatment weighting in observational research. Clin Kidney J 15:14–20. doi:10.1093/ckj/sfab158 PMC8757413 35035932

[23] Charlson ME, Pompei P, Ales KL, MacKenzie CR. 1987. A new method of classifying prognostic comorbidity in longitudinal studies: development and validation. J Chronic Dis 40:373–383. doi:10.1016/0021-9681(87)90171-8 3558716

[24] Lodise TP, Kanakamedala H, Hsu WC, Cai B. 2020. Impact of incremental delays in appropriate therapy on the outcomes of hospitalized adult patients with gram-negative bloodstream infections: “every day matters”. Pharmacotherapy 40:889–901. doi:10.1002/phar.2446 33112456

[25] Lodise TP, Patel N, Kwa A, Graves J, Furuno JP, Graffunder E, Lomaestro B, McGregor JC. 2007. Predictors of 30-day mortality among patients with *Pseudomonas aeruginosa* bloodstream infections: impact of delayed appropriate antibiotic selection. Antimicrob Agents Chemother 51:3510–3515. doi:10.1128/AAC.00338-07 PMC2043259 17646415

[26] Bonine NG, Berger A, Altincatal A, Wang R, Bhagnani T, Gillard P, Lodise T. 2019. Impact of delayed appropriate antibiotic therapy on patient outcomes by antibiotic resistance status from serious gram-negative bacterial infections. Am J Med Sci 357:103–110. doi:10.1016/j.amjms.2018.11.009 30665490

[27] Dang A, Thakker R, Li S, Hommel E, Mehta HB, Goodwin JS. 2022. Hospitalizations and mortality from non-SARS-CoV-2 causes among medicare beneficiaries at US hospitals during the SARS-CoV-2 pandemic. JAMA Netw Open 5:e221754. doi:10.1001/jamanetworkopen.2022.1754 PMC8908076 35262712

[28] Bravata DM, Perkins AJ, Myers LJ, Arling G, Zhang Y, Zillich AJ, Reese L, Dysangco A, Agarwal R, Myers J, Austin C, Sexson A, Leonard SJ, Dev S, Keyhani S. 2021. Association of intensive care unit patient load and demand with mortality rates in US department of veterans affairs hospitals during the COVID-19 pandemic. JAMA Netw Open 4:e2034266. doi:10.1001/jamanetworkopen.2020.34266 PMC7816100 33464319

[29] Jones BE, Ying J, Nevers MR, Alba PR, Patterson OV, Peterson KS, Rutter E, Christensen MA, Stern S, Jones MM, Gundlapalli A, Dean NC, Samore MC, Greene T. 2022. Trends in illness severity, hospitalization, and mortality for community-onset pneumonia at 118 US veterans affairs medical centers. J Gen Intern Med 37:3839–3847. doi:10.1007/s11606-022-07413-8 PMC8906522 35266121

[30] American Society of Health-System Pharmacists. 2022. Ceftolozane and tazobactam injection. Drug Shortages. University of Utah Drug Information Service. Available from: https://www.ashp.org/drug-shortages/current-shortages/drug-shortage-detail.aspx?id=705. Retrieved 30 Jul 2026.

[31] Vance MK, Cimino C, Ereshefsky B, Humphries R, Nelson GE. 2023. 2812. Clinical impact of the ceftolozane-tazobactam shortage on difficult-to-treat resistant *Pseudomonas aeruginosa* infections at an academic medical center. Open Forum Infect Dis 10. doi:10.1093/ofid/ofad500.2423

[32] Carpio BD, Dillon M, Hurtado D, VanDenberg T, Hayes JE, Carr AL, Minor SB. 2022. 1005. Financial and clinical impact of ceftolozane/tazobactam global recall on the treatment of multi-drug resistant *Pseudomonas* in a large health system. Open Forum Infect Dis 9. doi:10.1093/ofid/ofac492.846

[33] Chapman AB, Peterson KS, Rutter E, Nevers M, Zhang M, Ying J, Jones M, Classen D, Jones B. 2022. Development and evaluation of an interoperable natural language processing system for identifying pneumonia across clinical settings of care and institutions. JAMIA Open 5:ooac114. doi:10.1093/jamiaopen/ooac114 PMC9801965 36601365

[34] Jones BE, South BR, Shao Y, Lu CC, Leng J, Sauer BC, Gundlapalli AV, Samore MH, Zeng Q. 2018. Development and validation of a natural language processing tool to identify patients treated for pneumonia across VA emergency departments. Appl Clin Inform 9:122–128. doi:10.1055/s-0038-1626725 PMC5821510 29466818

[35] Cai M, Xie Y, Topol EJ, Al-Aly Z. 2024. Three-year outcomes of post-acute sequelae of COVID-19. Nat Med 30:1564–1573. doi:10.1038/s41591-024-02987-8 PMC11186764 38816608

[36] Shen K, Eddelbuettel JCP, Eisenberg MD. 2024. Job flows into and out of health care before and after the COVID-19 pandemic. JAMA Health Forum 5:e234964. doi:10.1001/jamahealthforum.2023.4964 PMC10818214 38277171

[37] Chawla N, Govier DJ, Than C, Rose D, Hynes DM, Hoggatt KJ, Yano EM. 2025. Chronic condition burden by veteran status, veterans health administration enrollment, and age using nationally representative survey data. Med Care 63:507–513. doi:10.1097/MLR.0000000000002159 40766989

[38] Vincent BM, Wiitala WL, Burns JA, Iwashyna TJ, Prescott HC. 2018. Using veterans affairs corporate data warehouse to identify 30-day hospital readmissions. Health Serv Outcomes Res Methodol 18:143–154. doi:10.1007/s10742-018-0178-3 PMC9518959 36176573

[39] Magiorakos AP, Srinivasan A, Carey RB, Carmeli Y, Falagas ME, Giske CG, Harbarth S, Hindler JF, Kahlmeter G, Olsson-Liljequist B, Paterson DL, Rice LB, Stelling J, Struelens MJ, Vatopoulos A, Weber JT, Monnet DL. 2012. Multidrug-resistant, extensively drug-resistant and pandrug-resistant bacteria: an international expert proposal for interim standard definitions for acquired resistance. Clin Microbiol Infect 18:268–281. doi:10.1111/j.1469-0691.2011.03570.x 21793988

[40] Centers for Disease Control and Prevention. 2022. Antimicrobial-resistant phenotype definitions: analysis of antimicrobial-resistant organisms in NHSN. Available from: https://www.cdc.gov/nhsn/pdfs/ps-analysis-resources/phenotype_definitions.pdf

[41] Talbot GH, Das A, Cush S, Dane A, Wible M, Echols R, Torres A, Cammarata S, Rex JH, Powers JH, Fleming T, Loutit J, Hoffmann S. 2019. Foundation for the National Institutes of health biomarkers consortium HVPT. J Infect Dis:1536–1544. doi:10.1093/infdis/jiy578

[42] Knirsch C, Alemayehu D, Botgros R, Comic-Savic S, Friedland D, Holland TL, Merchant K, Noel GJ, Pelfrene E, Reith C, Santiago J, Tiernan R, Tenearts P, Goldsack JC, Fowler VG Jr. 2016. Improving conduct and feasibility of clinical trials to evaluate antibacterial drugs to treat hospital-acquired bacterial pneumonia and ventilator-associated bacterial pneumonia: recommendations of the clinical trials transformation initiative antibacterial drug development project team. Clin Infect Dis 63 Suppl 2:S29–36. doi:10.1093/cid/ciw258 27481950

