# Supplementary material for: Comparative effectiveness of ceftolozane/tazobactam relative to ceftazidime/avibactam for the treatment of adult, hospitalized patients with pneumonia due to multidrug-resistant Pseudomonas aeruginosa in U.S. Veterans Health Administration facilities
Source: Antimicrob Agents Chemother. 2026 Sep 9;70(10):e00707-26. doi: 10.1128/aac.00707-26 (PMC13689478; doi:10.1128/aac.00707-26)
Supplement: Supplementary file 1 [file aac.00707-26-s0001.docx]

**Supplemental Figure 1. Love Plot for Covariate Balance Between Treatment Groups, Overall**

Abbreviation: IPTW, inverse probability of treatment weighting

**Supplemental Figure 2. Love Plot for Covariate Balance Between Treatment Groups, Overall, Alternative Model Incorporating Year of Infection and Geographic Region**

Abbreviation: IPTW, inverse probability of treatment weighting

**Supplemental Figure 3. Comparisons of Time to Multidrug-Resistant *Pseudomonas aeruginosa* Pneumonia Recurrence and Time to All-Cause Hospital Readmission Between Hospitalized Patients with Multidrug-Resistant *Pseudomonas aeruginosa* Pneumonia Treated with Ceftazidime/avibactam versus Ceftolozane/tazobactam**

Kaplan-Meier curves for MDR-PSA pneumonia recurrence (A) and all-cause hospital readmission (B). Patients who died before experiencing the outcome were censored at the time of death. Differences between treatment groups were evaluated using the log-rank test. Tick marks indicate censored observations. Numbers at risk are displayed below each panel.
Abbreviations: MDR-PSA, multidrug-resistant *Pseudomonas aeruginosa*; C/A, ceftazidime/avibactam; C/T, ceftolozane/tazobactam

**Supplemental Figure 4. Comparisons of Cumulative Incidence of Multidrug-Resistant *Pseudomonas aeruginosa* Pneumonia Recurrence and All-Cause Hospital Readmission Among Hospitalized Patients with Multidrug-Resistant *Pseudomonas aeruginosa* Pneumonia Treated with Ceftazidime/avibactam versus Ceftolozane/tazobactam, Accounting for the Competing Risk of Death**

Cumulative incidence functions for MDR-PSA pneumonia recurrence (A) and all-cause hospital readmission (B) were estimated using the Aalen-Johansen method, with death treated as a competing event. Differences in cumulative incidence between groups were evaluated using Gray’s test. Numbers at risk are displayed below each panel.
Abbreviations: MDR-PSA, multidrug-resistant *Pseudomonas aeruginosa*; C/A, ceftazidime/avibactam; C/T, ceftolozane/tazobactam

**Supplemental Figure 5. Love Plot for Covariate Balance Between Treatment Groups, Among Subgroup of Those With Early Treatment Within 72 Hours**

Abbreviation: IPTW, inverse probability of treatment weighting

**Supplemental Figure 6. Love Plot for Covariate Balance Between Treatment Groups, Among Subgroup of Those Without Sequential Therapy**

Abbreviation: IPTW, inverse probability of treatment weighting

| **Supplemental Table 1. IPTW-Weighted Baseline Characteristics of the Pseudopopulation of Hospitalized Patients with Multidrug-Resistant *Pseudomonas aeruginosa* Pneumonia Treated with Ceftazidime/avibactam or Ceftolozane/tazobactam** | | | |  |
| --- | --- | --- | --- | --- |
| **Characteristic** | **C/A**  (Weighted *n*=268) | **C/T**  (Weighted *n*=257) | **Absolute Weighted SMD** | |
| Age (years), median (Q1, Q3) | 70 (63, 77) | 69 (60, 75) | 0.167 | |
| Age ≥65 years, *n* (%) | 193 (72.1) | 174 (67.6) | 0.045 | |
| High complexity hospital, *n* (%) | 250 (93.3) | 233 (90.6) | 0.027 | |
| Geographic region, *n* (%) |  |  | 0.246 | |
| North Atlantic | 24 (9.0) | 49 (19.1) |  | |
| Southeast | 58 (21.5) | 103 (40.0) |  | |
| Midwest | 146 (54.6) | 77 (29.9) |  | |
| Continental | 40 (15.0) | 28 (11.0) |  | |
| Year of infection, *n* (%) |  |  | 0.087 | |
| 2015 | 0 (0.0) | 11 (4.2) |  | |
| 2016 | 19 (7.2) | 15 (5.7) |  | |
| 2017 | 22 (8.4) | 10 (3.8) |  | |
| 2018 | 46 (17.1) | 34 (13.2) |  | |
| 2019 | 34 (12.7) | 32 (12.4) |  | |
| 2022 | 60 (22.5) | 50 (19.6) |  | |
| 2023 | 23 (8.6) | 53 (20.8) |  | |
| 2024 | 63 (23.6) | 52 (20.2) |  | |
| Male, *n* (%) | 265 (98.9) | 252 (98.0) | 0.008 | |
| Obese, *n* (%) ^a^ | 52 (19.5) | 53 (20.5) | 0.010 | |
| Mechanically ventilated, *n* (%) | 196 (73.2) | 184 (71.6) | 0.016 | |
| ICU admission, *n* (%) | 196 (73.1) | 186 (72.5) | 0.006 | |
| Hospital length of stay prior to culture (hours), median (Q1, Q3) | 55 (6, 354) | 86 (3, 380) | 0.014 | |
| Hospital-acquired, *n* (%) ^b^ | 143 (53.4) | 137 (53.2) | 0.002 | |
| Hours from index culture to index treatment, median (Q1, Q3) | 46 (6, 97) | 52 (14, 74) | 0.078 | |
| Difficult-to-treat resistance (DTR) PSA, *n* (%) | 18 (6.9) | 32 (12.6) | 0.058 | |
| Carbapenem-resistant (CR) PSA, *n* (%) | 227 (84.6) | 222 (86.6) | 0.020 | |
| Polymicrobial pneumonia, any bacteria, *n* (%) | 79 (29.5) | 83 (32.3) | 0.028 | |
| Polymicrobial pneumonia, excluding *Staphylococcus* spp. and *Streptococcus* spp., *n* (%) | 76 (28.2) | 76 (29.5) | 0.014 | |
| SIRS criteria, *n* (%) |  |  | 0.054 | |
| 0 | 40 (15.0) | 33 (12.8) |  | |
| 1 | 87 (32.2) | 95 (36.8) |  | |
| 2 | 88 (32.8) | 71 (27.6) |  | |
| 3 | 48 (17.8) | 46 (17.7) |  | |
| 4 | 6 (2.2) | 13 (5.1) |  | |
| Sepsis, *n* (%) ^c^ | 210 (78.1) | 191 (74.4) | 0.037 | |
| Sepsis with evidence of tissue hypoperfusion, *n* (%) ^d^ | 31 (11.4) | 27 (10.4) | 0.010 | |
| Vasopressor requirement, *n* (%) | 85 (31.6) | 73 (28.3) | 0.032 | |
| Inodilator requirement, *n* (%) | 0 (0.0) | 2 (0.9) | 0.009 | |
| Creatinine clearance, *n* (%) |  |  | 0.011 | |
| >50 mL/min | 177 (66.1) | 172 (67.1) |  | |
| 30 – 50 mL/min | 40 (15.1) | 36 (14.0) |  | |
| 16 – 29 mL/min | 51 (18.8) | 48 (18.8) |  | |
| Acute kidney injury, *n* (%) ^e^ | 119 (44.3) | 101 (39.2) | 0.051 | |
| Hospitalization previous 90 days, *n* (%) | 142 (52.9) | 137 (53.4) | 0.005 | |
| Prior respiratory culture PSA positive during admission, *n* (%) ^f^ | 141 (52.7) | 133 (51.8) | 0.009 | |
| Prior blood culture PSA positive during admission, *n* (%) ^f^ | 18 (6.8) | 28 (11.0) | 0.043 | |
| Prior antibiotics received during admission, any, *n* (%) | 247 (92.0) | 242 (94.2) | 0.022 | |
| Prior aminoglycoside received during admission, *n* (%) | 92 (34.3) | 78 (30.5) | 0.038 | |
| Prior non-anti-PSA beta-lactam received during admission, *n* (%) | 120 (44.6) | 114 (44.5) | 0.001 | |
| Prior anti-PSA beta-lactam received during admission, *n* (%) | 234 (87.3) | 220 (85.6) | 0.018 | |
| Prior fluoroquinolone received during admission, *n* (%) | 87 (32.4) | 88 (34.4) | 0.020 | |
| Prior vancomycin received during admission, *n* (%) | 207 (77.3) | 199 (77.7) | 0.004 | |
| Prior daptomycin received during admission, *n* (%) | 29 (10.7) | 36 (14.0) | 0.033 | |
| Prior macrolide received during admission, *n* (%) | 52 (19.6) | 41 (15.8) | 0.037 | |
| Prior linezolid received during admission, *n* (%) | 26 (9.7) | 35 (13.8) | 0.041 | |
| Prior tetracycline received during admission, *n* (%) | 43 (16.0) | 12 (4.9) | 0.111 | |
| Prior sulfonamide received during admission, *n* (%) | 24 (9.0) | 35 (13.8) | 0.048 | |
| Prior polymyxin received during admission, *n* (%) | 24 (9.1) | 7 (2.7) | 0.064 | |
| Number of prior antibiotics received during admission, any, *n* (%) |  |  | 0.065 | |
| 0 | 21 (8.0) | 15 (5.8) |  | |
| 1 | 22 (8.3) | 32 (12.6) |  | |
| 2 | 26 (9.5) | 41 (16.0) |  | |
| 3 | 77 (28.9) | 59 (23.1) |  | |
| 4+ | 122 (45.3) | 109 (42.5) |  | |
| Concomitant antibiotics received, any, *n* (%) ^g^ | 244 (90.8) | 232 (90.2) | 0.007 | |
| Concomitant antibiotics received, anti-PSA, *n* (%) ^g^ | 215 (80.2) | 189 (73.8) | 0.064 | |
| Concomitant aminoglycoside received, *n* (%) ^g^ | 72 (26.7) | 63 (24.4) | 0.023 | |
| Concomitant non-anti-PSA beta-lactam received, *n* (%) ^g^ | 9 (3.3) | 36 (14.0) | 0.107 | |
| Concomitant anti-PSA beta-lactam received, *n* (%) ^g^ | 180 (67.1) | 151 (58.9) | 0.082 | |
| Concomitant fluoroquinolone received, *n* (%) ^g^ | 41 (15.3) | 40 (15.6) | 0.003 | |
| Concomitant vancomycin received, *n* (%) ^g^ | 158 (58.9) | 127 (49.4) | 0.095 | |
| Concomitant daptomycin received, *n* (%) ^g^ | 13 (4.7) | 16 (6.2) | 0.015 | |
| Concomitant macrolide received, *n* (%) ^g^ | 10 (3.7) | 8 (3.0) | 0.007 | |
| Concomitant linezolid received, *n* (%) ^g^ | 10 (3.8) | 19 (7.5) | 0.037 | |
| Concomitant tetracycline received, *n* (%) ^g^ | 12 (4.4) | 5 (2.1) | 0.022 | |
| Concomitant sulfonamide received, *n* (%) ^g^ | 11 (4.0) | 7 (2.9) | 0.011 | |
| Concomitant polymyxin received, *n* (%) ^g^ | 19 (7.2) | 5 (2.1) | 0.052 | |
| Number of concomitant antibiotics received, any, *n* (%) ^g^ |  |  | 0.067 | |
| 0 | 25 (9.2) | 25 (9.8) |  | |
| 1 | 63 (23.4) | 70 (27.2) |  | |
| 2 | 98 (36.5) | 105 (40.9) |  | |
| 3 | 58 (21.7) | 39 (15.0) |  | |
| 4+ | 25 (9.3) | 18 (7.1) |  | |
| Confirmed susceptibility to C/T, *n* (%) | 21 (7.9) | 14 (5.6) | 0.023 | |
| Confirmed susceptibility to C/A, *n* (%) | 12 (4.4) | 20 (7.7) | 0.033 | |
| Peptic ulcer disease, *n* (%) | 35 (13.0) | 39 (15.1) | 0.021 | |
| Rheumatologic disease, *n* (%) | 8 (2.9) | 11 (4.1) | 0.012 | |
| Chronic kidney disease, *n* (%) | 195 (72.6) | 213 (82.8) | 0.103 | |
| Peripheral vascular disease, *n* (%) | 94 (35.0) | 102 (39.6) | 0.046 | |
| Hemiplegia/paraplegia, *n* (%) | 103 (38.5) | 111 (43.4) | 0.049 | |
| Myocardial infarction, *n* (%) | 69 (25.8) | 69 (26.8) | 0.010 | |
| Malignancy, any, *n* (%) | 90 (33.7) | 63 (24.6) | 0.090 | |
| Metastatic solid tumor, *n* (%) | 19 (6.9) | 21 (8.1) | 0.011 | |
| Liver disease, mild, *n* (%) | 41 (15.1) | 54 (21.2) | 0.061 | |
| Liver disease, moderate-severe, *n* (%) | 36 (13.3) | 33 (12.8) | 0.004 | |
| Dementia, *n* (%) | 93 (34.8) | 60 (23.5) | 0.113 | |
| Diabetes, uncomplicated, *n* (%) | 103 (38.4) | 103 (40.1) | 0.017 | |
| Diabetes, complicated, *n* (%) | 96 (35.7) | 91 (35.6) | 0.001 | |
| Cerebrovascular disease, *n* (%) | 118 (44.1) | 95 (37.0) | 0.071 | |
| Chronic obstructive pulmonary disease, *n* (%) | 180 (67.0) | 165 (64.3) | 0.026 | |
| Congestive heart failure, *n* (%) | 148 (55.3) | 147 (57.3) | 0.020 | |
| Human immunodeficiency virus infection, *n* (%) | 4 (1.6) | 1 (0.5) | 0.012 | |
| Charlson comorbidity index, median (Q1, Q3) | 10.0  (7.0, 13.0) | 10.0  (7.0, 13.0) | 0.009 | |
| Modified APACHE II score, median (Q1, Q3) | 15.0  (11.0, 17.0) | 14.0  (10.0, 18.0) | 0.023 | |
| (Weighted event counts are shown rounded to the nearest whole number. Percentages are based on the IPTW-weighted pseudopopulation. Effective sample sizes after weighting were 109.31 for C/T and 93.19 for C/A. Continuous variables compared by Wilcoxon rank sum test and categorical variables compared by Pearson's Chi-squared test. Abbreviations: IPTW; inverse probability of treatment weighting; C/A; ceftazidime/avibactam; C/T; ceftolozane/tazobactam; SMD, standardized mean difference; ICU, intensive care unit; PSA, *Pseudomonas aeruginosa*; SIRS, systemic inflammatory response syndrome; APACHE, Acute Physiology and Chronic Health Evaluation; MDR-PSA, multidrug-resistant *Pseudomonas aeruginosa*; ICD-9/10-CM, International Classification of Diseases 9^th^/10^th^ Revision Clinical Modification ^a^ Body mass index ≥30 ^b^ MDR-PSA culture positive >48 hours after admission ^c^ SIRS ≥2 or ICD-9/10-CM diagnosis code for sepsis ^d^ Sepsis plus elevated lactate or systolic blood pressure <90 mmHg) ^e^ Determined by ICD-9/10-CM diagnosis code ^f^ Any culture positive for PSA collected before index MDR-PSA culture during the same hospitalization. Prior isolates were not required to meet MDR criteria or fulfill the study definition of pneumonia ^g^ Receipt of ≥1 dose during the period from first dose of C/T or C/A for index MDR-PSA pneumonia until the end of treatment with C/T or C/A | | | |  |

| Supplemental Table 2. IPTW-Weighted Clinical Outcomes in the Pseudopopulation of Hospitalized Patients with Multidrug-Resistant *Pseudomonas aeruginosa* Pneumonia Treated with Ceftazidime/avibactam or Ceftolozane/tazobactam | | |
| --- | --- | --- |
| Outcome | **C/A**  (Weighted *n*=268) | **C/T**  (Weighted *n*=257) |
| All-cause mortality, *n* (%) | | |
| 30-day mortality | 56 (20.7) | 54 (21.1) |
| 60-day mortality | 87 (32.4) | 83 (32.2) |
| 90-day mortality | 111 (41.5) | 113 (44.2) |
| MDR-PSA pneumonia recurrence, *n* (%) | | |
| 30-day recurrence | 59 (21.9) | 67 (26.1) |
| 60-day recurrence | 78 (29.2) | 82 (31.8) |
| 90-day recurrence | 83 (30.8) | 83 (32.4) |
| All-cause hospital readmission, *n* (%) | | |
| 30-day readmission | 38 (14.1) | 40 (15.5) |
| 60-day readmission | 72 (26.6) | 55 (21.5) |
| 90-day readmission | 76 (28.4) | 60 (23.5) |
| Clinical success at 30 days, *n* (%) ^a^ | 136 (50.5) | 110 (42.8) |

Weighted event counts are shown rounded to the nearest whole number. Percentages are based on the
IPTW-weighted pseudopopulation. Effective sample sizes after weighting were 109.31 for C/T and 93.19 for C/A.
Abbreviations: IPTW, inverse probability of treatment weighting; C/A; ceftazidime/avibactam;
C/T; ceftolozane/tazobactam; MDR-PSA, multidrug-resistant *Pseudomonas aeruginosa*
^a^ Composite of 30-day all-cause mortality, 30-day MDR-PSA pneumonia recurrence, and
30-day all-cause hospital readmission

| Supplemental Table 3. Comparison of Clinical Outcomes Between Hospitalized Patients with Multidrug-Resistant *Pseudomonas aeruginosa* Pneumonia Treated with Ceftazidime/avibactam versus Ceftolozane/tazobactam, Alternative Propensity Score Model | | |
| --- | --- | --- |
| Outcome (*N*=260) | **IPTW-Adjusted RR** *^a^* **(95% CI)** | ***P*** |
| All-cause mortality, *n* (%) | | |
| 30-day mortality | 0.83 (0.42-1.66) | 0.602 |
| 60-day mortality | 0.87 (0.53-1.40) | 0.562 |
| 90-day mortality | 0.88 (0.63-1.26) | 0.507 |
| MDR-PSA pneumonia recurrence, *n* (%) | | |
| 30-day recurrence | 1.28 (0.76-2.14) | 0.348 |
| 60-day recurrence | 1.09 (0.69-1.72) | 0.708 |
| 90-day recurrence | 1.02 (0.66-1.59) | 0.928 |
| All-cause hospital readmission, *n* (%) | | |
| 30-day readmission | 1.18 (0.63-2.24) | 0.598 |
| 60-day readmission | 0.93 (0.55-1.57) | 0.784 |
| 90-day readmission | 0.94 (0.57-1.55) | 0.804 |
| Clinical success at 30 days, *n* (%) ^b^ | 0.88 (0.63-1.24) | 0.465 |

Abbreviations: IPTW, inverse probability of treatment weighting; RR, risk ratio; CI, confidence interval; MDR-PSA, multidrug-resistant *Pseudomonas aeruginosa;* ICU, intensive care unit; SIRS, systemic inflammatory response syndrome; PSA, *Pseudomonas aeruginosa*; APACHE, Acute Physiology and Chronic Health Evaluation  *^a^* Propensity score models included the following variables: High complexity hospital, ICU admission, obesity, time to infection, vasopressor use, SIRS criteria, creatinine clearance category, prior PSA respiratory culture positive during admission, hospitalization in the previous 90 days, polymicrobial pneumonia, time to treatment, prior antibiotics during admission ≥ 4, Charlson comorbidity index, modified APACHE II score, year of infection, geographic region
^b^ Composite of 30-day all-cause mortality, 30-day MDR-PSA pneumonia recurrence, and 30-day all-cause hospital readmission

| **Supplemental Table 4. Time-Varying Association Between Treatment with Ceftazidime/avibactam versus Ceftolozane/tazobactam and Multidrug-Resistant *Pseudomonas aeruginosa* Pneumonia Recurrence in IPTW-Adjusted Cause-Specific Cox Regression** | | |
| --- | --- | --- |
| **Time** | **IPTW-Adjusted HR ^a^  (95% CI)** | ***P*** |
| Day 4 | 1.89 (1.03-3.47) | 0.040 |
| Day 7 | 1.49 (0.91-2.43) | 0.113 |
| Day 14 | 1.08 (0.69-1.69) | 0.746 |
| Day 30 | 0.74 (0.41-1.35) | 0.327 |
| Day 60 | 0.53 (0.23-1.20) | 0.126 |
| Day 90 | 0.43 (0.16-1.14) | 0.089 |

Time-varying treatment effect modeled using a treatment-by-log(time+1) interaction.
Time-specific adjusted HRs compare C/T with C/A, with adjusted HR >1 indicating
higher cause-specific hazard of MDR-PSA pneumonia recurrence with C/T relative to C/A.
Time-specific *P*-values represent pointwise Wald tests
Abbreviations: IPTW; inverse probability of treatment weighting; HR, hazard ratio;
CI, confidence interval; ICU, intensive care unit; SIRS, systemic inflammatory response
syndrome; PSA, *Pseudomonas aeruginosa*; APACHE, Acute Physiology and Chronic Health
Evaluation *^a^* Propensity score models included the following variables: High complexity hospital,
ICU admission, obesity, time to infection, vasopressor use, SIRS criteria, creatinine clearance
category, prior PSA respiratory culture positive during admission, hospitalization in the previous 90 days,
polymicrobial pneumonia, time to treatment, prior antibiotics during admission ≥ 4, Charlson
comorbidity index, modified APACHE II score

| **Supplemental Table 5. Comparison of Baseline Characteristics Between Hospitalized Patients with Multidrug-Resistant *Pseudomonas aeruginosa* Pneumonia Treated with Ceftazidime/avibactam versus Ceftolozane/tazobactam, Among Subgroup of Those With Early Treatment Within 72 Hours** | | | |
| --- | --- | --- | --- |
| **Characteristic** (*N*=175) | **C/A**  (*n*=89) | **C/T**  (*n*=86) | ***P*** |
| Age (years), median (Q1, Q3) | 70 (63, 76) | 69 (60, 76) | 0.318 |
| Age ≥65 years, *n* (%) | 62 (69.7) | 60 (69.8) | 0.988 |
| High complexity hospital, *n* (%) | 82 (92.1) | 81 (94.2) | 0.591 |
| Geographic region, *n* (%) |  |  | 0.622 |
| North Atlantic | 10 (11.2) | 13 (15.1) |  |
| Southeast | 25 (28.1) | 28 (32.6) |  |
| Midwest | 35 (39.3) | 32 (37.2) |  |
| Continental | 19 (21.3) | 13 (15.1) |  |
| Year of infection, *n* (%) |  |  | 0.050 |
| 2015 | 0 (0.0) | 4 (4.7) |  |
| 2016 | 6 (6.7) | 5 (5.8) |  |
| 2017 | 8 (9.0) | 3 (3.5) |  |
| 2018 | 18 (20.2) | 12 (14.0) |  |
| 2019 | 13 (14.6) | 15 (17.4) |  |
| 2022 | 21 (23.6) | 15 (17.4) |  |
| 2023 | 6 (6.7) | 17 (19.8) |  |
| 2024 | 17 (19.1) | 15 (17.4) |  |
| Male, *n* (%) | 87 (97.8) | 86 (100.0) | 0.162 |
| Obese, *n* (%) ^a^ | 16 (18.0) | 13 (15.1) | 0.611 |
| Mechanically ventilated, *n* (%) | 65 (73.0) | 65 (75.6) | 0.700 |
| ICU admission, *n* (%) | 60 (67.4) | 63 (73.3) | 0.398 |
| Hospital length of stay prior to culture (hours), median (Q1, Q3) | 86 (6, 330) | 108 (4, 478) | 0.630 |
| Hospital-acquired, *n* (%) ^b^ | 49 (55.1) | 49 (57.0) | 0.798 |
| Hours from index culture to index treatment, median (Q1, Q3) | 8 (0, 21) | 33 (10, 55) | <0.001 |
| Difficult-to-treat resistance (DTR) PSA, *n* (%) | 11 (12.4) | 11 (12.8) | 0.931 |
| Carbapenem-resistant (CR) PSA, *n* (%) | 77 (86.5) | 75 (87.2) | 0.892 |
| Polymicrobial pneumonia, any bacteria, *n* (%) | 34 (38.2) | 25 (29.1) | 0.201 |
| Polymicrobial pneumonia, excluding *Staphylococcus* spp. and *Streptococcus* spp., *n* (%) | 32 (36.0) | 23 (26.7) | 0.189 |
| SIRS criteria, *n* (%) |  |  | 0.630 |
| 0 | 12 (13.5) | 9 (10.5) |  |
| 1 | 31 (34.8) | 34 (39.5) |  |
| 2 | 25 (28.1) | 24 (27.9) |  |
| 3 | 19 (21.3) | 14 (16.3) |  |
| 4 | 2 (2.2) | 5 (5.8) |  |
| Sepsis, *n* (%) ^c^ | 66 (74.2) | 64 (74.4) | 0.968 |
| Sepsis with evidence of tissue hypoperfusion, *n* (%) ^d^ | 16 (18.0) | 12 (14.0) | 0.468 |
| Vasopressor requirement, *n* (%) | 25 (28.1) | 26 (30.2) | 0.755 |
| Inodilator requirement, *n* (%) | 0 (0.0) | 1 (1.2) | 0.308 |
| Creatinine clearance, *n* (%) |  |  | 0.517 |
| >50 mL/min | 60 (67.4) | 55 (64.0) |  |
| 30 – 50 mL/min | 12 (13.5) | 17 (19.8) |  |
| 16 – 29 mL/min | 17 (19.1) | 14 (16.3) |  |
| Acute kidney injury, *n* (%) ^e^ | 37 (41.6) | 39 (45.3) | 0.614 |
| Hospitalization previous 90 days, *n* (%) | 40 (44.9) | 52 (60.5) | 0.040 |
| Prior respiratory culture PSA positive during admission, *n* (%) ^f^ | 43 (48.3) | 58 (67.4) | 0.010 |
| Prior blood culture PSA positive during admission, *n* (%) ^f^ | 7 (7.9) | 12 (14.0) | 0.196 |
| Prior antibiotics received during admission, any, *n* (%) | 76 (85.4) | 82 (95.3) | 0.026 |
| Prior aminoglycoside received during admission, *n* (%) | 28 (31.5) | 28 (32.6) | 0.876 |
| Prior non-anti-PSA beta-lactam received during admission, *n* (%) | 37 (41.6) | 45 (52.3) | 0.154 |
| Prior anti-PSA beta-lactam received during admission, *n* (%) | 67 (75.3) | 77 (89.5) | 0.014 |
| Prior fluoroquinolone received during admission, *n* (%) | 22 (24.7) | 34 (39.5) | 0.036 |
| Prior vancomycin received during admission, *n* (%) | 62 (69.7) | 67 (77.9) | 0.215 |
| Prior daptomycin received during admission, *n* (%) | 9 (10.1) | 12 (14.0) | 0.434 |
| Prior macrolide received during admission, *n* (%) | 10 (11.2) | 14 (16.3) | 0.332 |
| Prior linezolid received during admission, *n* (%) | 12 (13.5) | 11 (12.8) | 0.892 |
| Prior tetracycline received during admission, *n* (%) | 7 (7.9) | 6 (7.0) | 0.823 |
| Prior sulfonamide received during admission, *n* (%) | 9 (10.1) | 13 (15.1) | 0.318 |
| Prior polymyxin received during admission, *n* (%) | 6 (6.7) | 3 (3.5) | 0.330 |
| Number of prior antibiotics received during admission, any, *n* (%) |  |  | 0.082 |
| 0 | 13 (14.6) | 4 (4.7) |  |
| 1 | 10 (11.2) | 8 (9.3) |  |
| 2 | 8 (9.0) | 14 (16.3) |  |
| 3 | 24 (27.0) | 18 (20.9) |  |
| 4+ | 34 (38.2) | 42 (48.8) |  |
| Concomitant antibiotics received, any, *n* (%) ^g^ | 78 (87.6) | 76 (88.4) | 0.882 |
| Concomitant antibiotics received, anti-PSA, *n* (%) ^g^ | 62 (69.7) | 62 (72.1) | 0.724 |
| Concomitant aminoglycoside received, *n* (%) ^g^ | 17 (19.1) | 18 (20.9) | 0.762 |
| Concomitant non-anti-PSA beta-lactam received, *n* (%) ^g^ | 6 (6.7) | 13 (15.1) | 0.075 |
| Concomitant anti-PSA beta-lactam received, *n* (%) ^g^ | 49 (55.1) | 45 (52.3) | 0.717 |
| Concomitant fluoroquinolone received, *n* (%) ^g^ | 13 (14.6) | 17 (19.8) | 0.365 |
| Concomitant vancomycin received, *n* (%) ^g^ | 50 (56.2) | 41 (47.7) | 0.260 |
| Concomitant daptomycin received, *n* (%) ^g^ | 7 (7.9) | 5 (5.8) | 0.591 |
| Concomitant macrolide received, *n* (%) ^g^ | 2 (2.2) | 1 (1.2) | 0.581 |
| Concomitant linezolid received, *n* (%) ^g^ | 6 (6.7) | 7 (8.1) | 0.724 |
| Concomitant tetracycline received, *n* (%) ^g^ | 5 (5.6) | 2 (2.3) | 0.267 |
| Concomitant sulfonamide received, *n* (%) ^g^ | 3 (3.4) | 3 (3.5) | 0.966 |
| Concomitant polymyxin received, *n* (%) ^g^ | 3 (3.4) | 2 (2.3) | 0.678 |
| Number of concomitant antibiotics received, any, *n* (%) ^g^ |  |  | 0.939 |
| 0 | 11 (12.4) | 10 (11.6) |  |
| 1 | 24 (27.0) | 24 (27.9) |  |
| 2 | 32 (36.0) | 35 (40.7) |  |
| 3 | 16 (18.0) | 12 (14.0) |  |
| 4+ | 6 (6.7) | 5 (5.8) |  |
| Confirmed susceptibility to C/T, *n* (%) | 6 (6.7) | 4 (4.7) | 0.551 |
| Confirmed susceptibility to C/A, *n* (%) | 1 (1.1) | 4 (4.7) | 0.161 |
| Peptic ulcer disease, *n* (%) | 16 (18.0) | 14 (16.3) | 0.766 |
| Rheumatologic disease, *n* (%) | 2 (2.2) | 5 (5.8) | 0.229 |
| Chronic kidney disease, *n* (%) | 63 (70.8) | 78 (90.7) | <0.001 |
| Peripheral vascular disease, *n* (%) | 26 (29.2) | 37 (43.0) | 0.057 |
| Hemiplegia/paraplegia, *n* (%) | 39 (43.8) | 38 (44.2) | 0.961 |
| Myocardial infarction, *n* (%) | 24 (27.0) | 24 (27.9) | 0.889 |
| Malignancy, any, *n* (%) | 25 (28.1) | 24 (27.9) | 0.979 |
| Metastatic solid tumor, *n* (%) | 7 (7.9) | 10 (11.6) | 0.401 |
| Liver disease, mild, *n* (%) | 15 (16.9) | 23 (26.7) | 0.113 |
| Liver disease, moderate-severe, *n* (%) | 11 (12.4) | 14 (16.3) | 0.459 |
| Dementia, *n* (%) | 27 (30.3) | 21 (24.4) | 0.380 |
| Diabetes, uncomplicated, *n* (%) | 39 (43.8) | 37 (43.0) | 0.915 |
| Diabetes, complicated, *n* (%) | 32 (36.0) | 37 (43.0) | 0.339 |
| Cerebrovascular disease, *n* (%) | 38 (42.7) | 32 (37.2) | 0.459 |
| Chronic obstructive pulmonary disease, *n* (%) | 62 (69.7) | 61 (70.9) | 0.854 |
| Congestive heart failure, *n* (%) | 38 (42.7) | 52 (60.5) | 0.019 |
| Human immunodeficiency virus infection, *n* (%) | 1 (1.1) | 0 (0.0) | 0.324 |
| Charlson comorbidity index, median (Q1, Q3) | 9.0  (7.0, 12.0) | 11.0  (8.0, 14.0) | 0.044 |
| Modified APACHE II score, median (Q1, Q3) | 14.0  (10.0, 17.0) | 15.0  (11.0, 18.0) | 0.207 |
| (Continuous variables compared by Wilcoxon rank sum test and categorical variables compared by Pearson's Chi-squared test or Fisher’s exact test. Abbreviations: C/A; ceftazidime/avibactam; C/T; ceftolozane/tazobactam; ICU, intensive care unit; PSA, *Pseudomonas aeruginosa*; SIRS, systemic inflammatory response syndrome; APACHE, Acute Physiology and Chronic Health Evaluation; MDR-PSA, multidrug-resistant *Pseudomonas aeruginosa*; ICD-9/10-CM, International Classification of Diseases 9^th^/10^th^ Revision Clinical Modification ^a^ Body mass index ≥30 ^b^ MDR-PSA culture positive >48 hours after admission ^c^ SIRS ≥2 or ICD-9/10-CM diagnosis code for sepsis ^d^ Sepsis plus elevated lactate or systolic blood pressure <90 mmHg) ^e^ Determined by ICD-9/10-CM diagnosis code ^f^ Any culture positive for PSA collected before index MDR-PSA culture during the same hospitalization. Prior isolates were not required to meet MDR criteria or fulfill the study definition of pneumonia ^g^ Receipt of ≥1 dose during the period from first dose of C/T or C/A for index MDR-PSA pneumonia until the end of treatment with C/T or C/A | | | |

| Supplemental Table 6. Comparison of Clinical Outcomes Between Hospitalized Patients with Multidrug-Resistant *Pseudomonas aeruginosa* Pneumonia Treated with Ceftazidime/avibactam versus Ceftolozane/tazobactam, Among Subgroup of Those With Early Treatment Within 72 Hours | | | | | | |
| --- | --- | --- | --- | --- | --- | --- |
| Outcome  (*N*=175) | **C/A** (*n*=89) | **C/T**  (*n*=86) | **Unadjusted RR  (95% CI)** | ***P*** | **IPTW-Adjusted RR** *^a^* **(95% CI)** | ***P*** |
| All-cause mortality, *n* (%) | | | | | | |
| 30-day mortality | 9  (10.1) | 18 (20.9) | 2.07  (0.97-4.38) | 0.057 | 1.18  (0.47-2.93) | 0.724 |
| 60-day mortality | 22 (24.7) | 29 (33.7) | 1.36  (0.85-2.19) | 0.197 | 1.15  (0.61-2.16) | 0.666 |
| 90-day mortality | 33 (37.1) | 39 (45.3) | 1.22  (0.85-1.75) | 0.271 | 0.92  (0.57-1.48) | 0.724 |
| MDR-PSA pneumonia recurrence, *n* (%) | | | | | | |
| 30-day recurrence | 27 (30.3) | 24 (27.9) | 0.92  (0.58-1.47) | 0.725 | 0.86  (0.41-1.79) | 0.680 |
| 60-day recurrence | 33 (37.1) | 29 (33.7) | 0.91  (0.61-1.36) | 0.644 | 0.78  (0.45-1.37) | 0.381 |
| 90-day recurrence | 35 (39.3) | 30 (34.9) | 0.89  (0.60-1.31) | 0.546 | 0.77  (0.45-1.32) | 0.341 |
| All-cause hospital readmission, *n* (%) | | | | | | |
| 30-day readmission | 20 (22.5) | 14 (16.3) | 0.72  (0.39-1.35) | 0.307 | 0.95 (0.45-2.06) | 0.903 |
| 60-day readmission | 25 (28.1) | 18 (20.9) | 0.75  (0.44-1.27) | 0.278 | 0.94 (0.47-1.86) | 0.865 |
| 90-day readmission | 28 (31.6) | 19 (22.1) | 0.70  (0.42-1.17) | 0.170 | 0.90 (0.46-1.74) | 0.746 |
| Clinical success at 30 days, *n* (%) ^b^ | 45 (50.6) | 36 (41.9) | 0.83  (0.59-1.15) | 0.255 | 0.98  (0.59-1.64) | 0.948 |
| Hospital length of stay (days), median, IQR ^c^ | 22  (11-57) | 24.5 (14-60) | --- | 0.459 | --- | --- |
| ICU-free days, median (IQR) ^c,d^ | 18  (2-28) | 13  (0-29) | --- | 0.256 | --- | --- |

Abbreviations: C/A; ceftazidime/avibactam; C/T; ceftolozane/tazobactam; RR, risk ratio; CI, confidence interval; IPTW, inverse probability of treatment weighting; MDR-PSA, multidrug-resistant *Pseudomonas aeruginosa*; IQR, interquartile range; ICU, intensive care unit; SIRS, systemic inflammatory response syndrome; PSA, *Pseudomonas aeruginosa*; APACHE, Acute Physiology and Chronic Health Evaluation *^a^* Propensity score models included the following variables: High complexity hospital, ICU admission, obesity, time to infection, vasopressor use, SIRS criteria, creatinine clearance category, prior PSA respiratory culture positive during admission, hospitalization in the previous 90 days, polymicrobial pneumonia, time to treatment, prior antibiotics during admission ≥ 4, Charlson comorbidity index, modified APACHE II score
^b^ Composite of 30-day all-cause mortality, 30-day MDR-PSA pneumonia recurrence, and 30-day all-cause hospital readmission
^c^ Compared by Wilcoxon rank sum test
^d^ Within 30 days from start of treatment

| **Supplemental Table 7. Comparison of Baseline Characteristics Between Hospitalized Patients with Multidrug-Resistant *Pseudomonas aeruginosa* Pneumonia Treated with Ceftazidime/avibactam versus Ceftolozane/tazobactam, Among Subgroup of Those Without Sequential Therapy** | | | |
| --- | --- | --- | --- |
| **Characteristic** (*N*=233) | **C/A**  (*n*=121) | **C/T**  (*n*=112) | ***P*** |
| Age (years), median (Q1, Q3) | 69 (63, 76) | 70 (61, 75) | 0.537 |
| Age ≥65 years, *n* (%) | 84 (69.4) | 80 (71.4) | 0.737 |
| High complexity hospital, *n* (%) | 110 (90.9) | 108 (96.4) | 0.086 |
| Geographic region, *n* (%) |  |  | 0.008 |
| North Atlantic | 12 (9.9) | 18 (16.1) |  |
| Southeast | 28 (23.1) | 44 (39.3) |  |
| Midwest | 61 (50.4) | 37 (33.0) |  |
| Continental | 20 (16.5) | 13 (11.6) |  |
| Year of infection, *n* (%) |  |  | 0.018 |
| 2015 | 0 (0.0) | 5 (4.5) |  |
| 2016 | 8 (6.6) | 6 (5.4) |  |
| 2017 | 12 (9.9) | 4 (3.6) |  |
| 2018 | 21 (17.4) | 15 (13.4) |  |
| 2019 | 19 (15.7) | 15 (13.4) |  |
| 2022 | 25 (20.7) | 21 (18.8) |  |
| 2023 | 10 (8.3) | 24 (21.4) |  |
| 2024 | 26 (21.5) | 22 (19.6) |  |
| Male, *n* (%) | 119 (98.3) | 110 (98.2) | 0.938 |
| Obese, *n* (%) ^a^ | 24 (19.8) | 20 (17.9) | 0.700 |
| Mechanically ventilated, *n* (%) | 86 (71.1) | 84 (75.0) | 0.500 |
| ICU admission, *n* (%) | 84 (69.4) | 83 (74.1) | 0.428 |
| Hospital length of stay prior to culture (hours), median (Q1, Q3) | 55  (6, 323) | 96  (1, 374) | 0.932 |
| Hospital-acquired, *n* (%) ^b^ | 65 (53.7) | 61 (54.5) | 0.909 |
| Hours from index culture to index treatment, median (Q1, Q3) | 19 (0, 77) | 59 (28, 80) | <0.001 |
| Difficult-to-treat resistance (DTR) PSA, *n* (%) | 10 (8.3) | 13 (11.6) | 0.393 |
| Carbapenem-resistant (CR) PSA, *n* (%) | 103 (85.1) | 95 (84.8) | 0.949 |
| Polymicrobial pneumonia, any bacteria, *n* (%) | 39 (32.2) | 29 (25.9) | 0.288 |
| Polymicrobial pneumonia, excluding *Staphylococcus* spp. and *Streptococcus* spp., *n* (%) | 38 (31.4) | 26 (23.2) | 0.162 |
| SIRS criteria, *n* (%) |  |  | 0.824 |
| 0 | 17 (14.0) | 18 (16.1) |  |
| 1 | 41 (33.9) | 44 (39.3) |  |
| 2 | 35 (28.9) | 30 (26.8) |  |
| 3 | 25 (20.7) | 18 (16.1) |  |
| 4 | 3 (2.5) | 2 (1.8) |  |
| Sepsis, *n* (%) ^c^ | 91 (75.2) | 80 (71.4) | 0.514 |
| Sepsis with evidence of tissue hypoperfusion, *n* (%) ^d^ | 18 (14.9) | 10 (8.9) | 0.163 |
| Vasopressor requirement, *n* (%) | 37 (30.6) | 30 (26.8) | 0.523 |
| Inodilator requirement, *n* (%) | 0 (0.0) | 1 (0.9) | 0.298 |
| Creatinine clearance, *n* (%) |  |  | 0.280 |
| >50 mL/min | 82 (67.8) | 75 (67.0) |  |
| 30 – 50 mL/min | 13 (10.7) | 19 (17.0) |  |
| 16 – 29 mL/min | 26 (21.5) | 18 (16.1) |  |
| Acute kidney injury, *n* (%) ^e^ | 50 (41.3) | 47 (42.0) | 0.921 |
| Hospitalization previous 90 days, *n* (%) | 61 (50.4) | 66 (58.9) | 0.192 |
| Prior respiratory culture PSA positive during admission, *n* (%) ^f^ | 50 (41.3) | 62 (55.4) | 0.032 |
| Prior blood culture PSA positive during admission, *n* (%) ^f^ | 7 (5.8) | 8 (7.1) | 0.673 |
| Prior antibiotics received during admission, any, *n* (%) | 106 (87.6) | 106 (94.6) | 0.061 |
| Prior aminoglycoside received during admission, *n* (%) | 32 (26.4) | 29 (25.9) | 0.924 |
| Prior non-anti-PSA beta-lactam received during admission, *n* (%) | 46 (38.0) | 54 (48.2) | 0.116 |
| Prior anti-PSA beta-lactam received during admission, *n* (%) | 97 (80.2) | 102 (91.1) | 0.018 |
| Prior fluoroquinolone received during admission, *n* (%) | 30 (24.8) | 39 (34.8) | 0.094 |
| Prior vancomycin received during admission, *n* (%) | 87 (71.9) | 84 (75.0) | 0.593 |
| Prior daptomycin received during admission, *n* (%) | 11 (9.1) | 16 (14.3) | 0.216 |
| Prior macrolide received during admission, *n* (%) | 21 (17.4) | 20 (17.9) | 0.920 |
| Prior linezolid received during admission, *n* (%) | 11 (9.1) | 16 (14.3) | 0.216 |
| Prior tetracycline received during admission, *n* (%) | 14 (11.6) | 7 (6.3) | 0.157 |
| Prior sulfonamide received during admission, *n* (%) | 13 (10.7) | 16 (14.3) | 0.413 |
| Prior polymyxin received during admission, *n* (%) | 9 (7.4) | 2 (1.8) | 0.042 |
| Number of prior antibiotics received during admission, any, *n* (%) |  |  | 0.279 |
| 0 | 15 (12.4) | 6 (5.4) |  |
| 1 | 14 (11.6) | 11 (9.8) |  |
| 2 | 13 (10.7) | 19 (17.0) |  |
| 3 | 32 (26.4) | 30 (26.8) |  |
| 4+ | 47 (38.8) | 46 (41.1) |  |
| Concomitant antibiotics received, any, *n* (%) ^g^ | 107 (88.4) | 100 (89.3) | 0.836 |
| Concomitant antibiotics received, anti-PSA, *n* (%) ^g^ | 90 (74.4) | 91 (81.3) | 0.208 |
| Concomitant aminoglycoside received, *n* (%) ^g^ | 28 (23.1) | 24 (21.4) | 0.754 |
| Concomitant non-anti-PSA beta-lactam received, *n* (%) ^g^ | 5 (4.1) | 16 (14.3) | 0.007 |
| Concomitant anti-PSA beta-lactam received, *n* (%) ^g^ | 76 (62.8) | 78 (69.6) | 0.271 |
| Concomitant fluoroquinolone received, *n* (%) ^g^ | 16 (13.2) | 16 (14.3) | 0.814 |
| Concomitant vancomycin received, *n* (%) ^g^ | 64 (52.9) | 49 (43.8) | 0.163 |
| Concomitant daptomycin received, *n* (%) ^g^ | 7 (5.8) | 8 (7.1) | 0.673 |
| Concomitant macrolide received, *n* (%) ^g^ | 4 (3.3) | 5 (4.5) | 0.647 |
| Concomitant linezolid received, *n* (%) ^g^ | 6 (5.0) | 9 (8.0) | 0.339 |
| Concomitant tetracycline received, *n* (%) ^g^ | 7 (5.8) | 1 (0.9) | 0.040 |
| Concomitant sulfonamide received, *n* (%) ^g^ | 6 (5.0) | 3 (2.7) | 0.367 |
| Concomitant polymyxin received, *n* (%) ^g^ | 6 (5.0) | 2 (1.8) | 0.184 |
| Number of concomitant antibiotics received, any, *n* (%) ^g^ |  |  | 0.800 |
| 0 | 14 (11.6) | 12 (10.7) |  |
| 1 | 33 (27.3) | 27 (24.1) |  |
| 2 | 41 (33.9) | 47 (42.0) |  |
| 3 | 23 (19.0) | 18 (16.1) |  |
| 4+ | 10 (8.3) | 8 (7.1) |  |
| Confirmed susceptibility to C/T, *n* (%) | 9 (7.4) | 6 (5.4) | 0.518 |
| Confirmed susceptibility to C/A, *n* (%) | 4 (3.3) | 5 (4.5) | 0.647 |
| Peptic ulcer disease, *n* (%) | 17 (14.0) | 15 (13.4) | 0.884 |
| Rheumatologic disease, *n* (%) | 4 (3.3) | 4 (3.6) | 0.911 |
| Chronic kidney disease, *n* (%) | 85 (70.2) | 91 (81.3) | 0.051 |
| Peripheral vascular disease, *n* (%) | 35 (28.9) | 43 (38.4) | 0.126 |
| Hemiplegia/paraplegia, *n* (%) | 45 (37.2) | 45 (40.2) | 0.640 |
| Myocardial infarction, *n* (%) | 31 (25.6) | 27 (24.1) | 0.790 |
| Malignancy, any, *n* (%) | 37 (30.6) | 32 (28.6) | 0.737 |
| Metastatic solid tumor, *n* (%) | 9 (7.4) | 13 (11.6) | 0.277 |
| Liver disease, mild, *n* (%) | 17 (14.0) | 23 (20.5) | 0.190 |
| Liver disease, moderate-severe, *n* (%) | 16 (13.2) | 13 (11.6) | 0.709 |
| Dementia, *n* (%) | 37 (30.6) | 28 (25.0) | 0.343 |
| Diabetes, uncomplicated, *n* (%) | 47 (38.8) | 45 (40.2) | 0.835 |
| Diabetes, complicated, *n* (%) | 43 (35.5) | 40 (35.7) | 0.977 |
| Cerebrovascular disease, *n* (%) | 48 (39.7) | 39 (34.8) | 0.445 |
| Chronic obstructive pulmonary disease, *n* (%) | 82 (67.8) | 76 (67.9) | 0.988 |
| Congestive heart failure, *n* (%) | 58 (47.9) | 63 (56.3) | 0.204 |
| Human immunodeficiency virus infection, *n* (%) | 2 (1.7) | 0 (0.0) | 0.172 |
| Charlson comorbidity index, median (Q1, Q3) | 9.0  (7.0, 12.0) | 10.0  (7.5, 13.0) | 0.183 |
| Modified APACHE II score, median (Q1, Q3) | 14.0  (11.0, 17.0) | 14.0  (11.5, 17.5) | 0.658 |
| (Continuous variables compared by Wilcoxon rank sum test and categorical variables compared by Pearson's Chi-squared test or Fisher’s exact test. Abbreviations: C/A; ceftazidime/avibactam; C/T; ceftolozane/tazobactam; ICU, intensive care unit; PSA, *Pseudomonas aeruginosa*; SIRS, systemic inflammatory response syndrome; APACHE, Acute Physiology and Chronic Health Evaluation; MDR-PSA, multidrug-resistant *Pseudomonas aeruginosa*; ICD-9/10-CM, International Classification of Diseases 9^th^/10^th^ Revision Clinical Modification ^a^ Body mass index ≥30 ^b^ MDR-PSA culture positive >48 hours after admission ^c^ SIRS ≥2 or ICD-9/10-CM diagnosis code for sepsis ^d^ Sepsis plus elevated lactate or systolic blood pressure <90 mmHg) ^e^ Determined by ICD-9/10-CM diagnosis code ^f^ Any culture positive for PSA collected before index MDR-PSA culture during the same hospitalization. Prior isolates were not required to meet MDR criteria or fulfill the study definition of pneumonia ^g^ Receipt of ≥1 dose during the period from first dose of C/T or C/A for index MDR-PSA pneumonia until the end of treatment with C/T or C/A | | | |

| Supplemental Table 8. Comparison of Clinical Outcomes Between Hospitalized Patients with Multidrug-Resistant *Pseudomonas aeruginosa* Pneumonia Treated with Ceftazidime/avibactam versus Ceftolozane/tazobactam, Among Subgroup of Those Without Sequential Therapy | | | | | | |
| --- | --- | --- | --- | --- | --- | --- |
| Outcome  (*N*=233) | **C/A** (*n*=121) | **C/T**  (*n*=112) | **Unadjusted RR  (95% CI)** | ***P*** | **IPTW-Adjusted RR** *^a^* **(95% CI)** | ***P*** |
| All-cause mortality, *n* (%) | | | | | | |
| 30-day mortality | 20 (16.5) | 27 (24.1) | 1.46 (0.87-2.45) | 0.156 | 1.11 (0.61-2.04) | 0.727 |
| 60-day mortality | 36 (29.8) | 41 (36.6) | 1.23 (0.85-1.78) | 0.270 | 1.09 (0.72-1.67) | 0.672 |
| 90-day mortality | 50 (41.3) | 53 (47.3) | 1.14 (0.86-1.53) | 0.359 | 1.15 (0.83-1.60) | 0.410 |
| MDR-PSA pneumonia recurrence, *n* (%) | | | | | | |
| 30-day recurrence | 29 (24.0) | 28 (25.0) | 1.04 (0.66-1.64) | 0.855 | 1.17 (0.70-1.95) | 0.533 |
| 60-day recurrence | 37 (30.6) | 34 (30.4) | 0.99 (0.67-1.47) | 0.971 | 1.06 (0.68-1.64) | 0.796 |
| 90-day recurrence | 39 (32.2) | 35 (31.2) | 0.97 (0.66-1.42) | 0.873 | 1.02 (0.67-1.57) | 0.917 |
| All-cause hospital readmission, *n* (%) | | | | | | |
| 30-day readmission | 21 (17.4) | 18 (16.1) | 0.93 (0.52-1.65) | 0.794 | 1.09  (0.58-2.04) | 0.783 |
| 60-day readmission | 32 (26.4) | 26 (23.2) | 0.88  (0.56-1.38) | 0.571 | 0.81 (0.49-1.34) | 0.415 |
| 90-day readmission | 34 (28.1) | 29 (25.9) | 0.92 (0.60-1.41) | 0.706 | 0.86 (0.53-1.38) | 0.535 |
| Clinical success at 30 days, *n* (%) ^b^ | 62 (51.2) | 46 (41.1) | 0.80 (0.60-1.06) | 0.126 | 0.82 (0.59-1.13) | 0.227 |
| Hospital length of stay (days), median, IQR ^c^ | 22  (11-46) | 28  (14-59) | --- | 0.122 | --- | --- |
| ICU-free days, median (IQR) ^c,d^ | 19.2 (0-28) | 10.3 (0-29) | --- | 0.088 | --- | --- |

Abbreviations: C/A; ceftazidime/avibactam; C/T; ceftolozane/tazobactam; RR, risk ratio; CI, confidence interval; IPTW, inverse probability of treatment weighting; PSA, *Pseudomonas aeruginosa*; MDR-PSA, multidrug-resistant *Pseudomonas aeruginosa*; IQR, interquartile range; ICU, intensive care unit; SIRS, systemic inflammatory response syndrome; APACHE, Acute Physiology and Chronic Health Evaluation *^a^* Propensity score models included the following variables: High complexity hospital, ICU admission, obesity, time to infection, vasopressor use, SIRS criteria, creatinine clearance category, prior PSA respiratory culture positive during admission, hospitalization in the previous 90 days, polymicrobial pneumonia, time to treatment, prior antibiotics during admission ≥ 4, Charlson comorbidity index, modified APACHE II score
^b^ Composite of 30-day all-cause mortality, 30-day MDR-PSA pneumonia recurrence, and 30-day all-cause hospital readmission
^c^ Compared by Wilcoxon rank sum test
^d^ Within 30 days from start of treatment
